# Design, synthesis and medical prospects of electrospun molecularly imprinted fibers

**DOI:** 10.1038/s41598-025-11114-7

**Published:** 2025-07-18

**Authors:** Sarah H. Megahed, Mohammad Abdel-Halim, Yahia I. El-shabrawy, Engy M. Saad, Amr Hefnawy, Heba Handoussa, Boris Mizaikoff, Nesrine A. El Gohary

**Affiliations:** 1 Pharmaceutical Chemistry Department, Faculty of Pharmacy and Biotechnology, German University in Cairo, Cairo, 11835 Egypt; 2 Microbiology and Immunology Department, Faculty of Pharmacy and Biotechnology, German University in Cairo, Cairo, 11835 Egypt; 3 Division of Molecular Pharmaceutics and Drug Delivery, College of Pharmacy, University of Texas at Austin, Austin, TX USA; 4 Pharmaceutical Biology Department, Faculty of Pharmacy and Biotechnology, German University in Cairo, Cairo, 11835 Egypt; 5 Institute of Analytical and Bioanalytical Chemistry, Ulm University, 89081 Ulm, Germany; 6 Hahn-Schickard, 89077 Ulm, Germany

**Keywords:** Molecularly imprinted fibers, Ferulic acid, Khellin, Electrospinning, Polycaprolactone, Design of experiments, Drug delivery, Biotechnology, Nanostructures, Bioinspired materials, Polymers, Vitiligo

## Abstract

The current study describes fabrication of molecularly imprinted (MI) electrospun nanofibers of average diameter 500 nm for controlled delivery of two templates; ferulic acid (FA) and khellin. Polycaprolactone (PCL) was used as supporting matrix in fiber preparation while polyallylamine (PAM) was added to provide molecular recognition sites for the template. The preparation of MI fibers was optimized using design of experiments (DoE). For FA fibers, a fractional factorial deign was used to screen different factors involved in the electrospinning process and it was found that FA and PAM concentration; in addition to PCL concentration were significant. These two variables were used to create a central composite design to optimize fiber diameter. Control fibers were prepared by directly loading ferulic acid into the fiber matrix without PAM. In vitro release studies revealed that MI fibers had more controlled release of FA compared to control fibers. DoE were also used to optimize the diameter of khellin MI fibers. In vitro release studies for khellin MI fibers did not show any significant difference when compared to control fibers. MTT assay was conducted to assess the cytotoxicity of MI fibers against mouse melanoma B16F10 cell lines. FA fibers were well-tolerated by the cells up to a concentration of 250 µg/ml, while khellin fibers showed no cytotoxicity up to a concentration of 125 µg/ml. On the other hand, the non-medicated fibers prepared with only PCL/PAM were safe up to 500 µg/ml, suggesting that the cytotoxicity observed at higher concentration of the MI fibers is due to the effect of FA or khellin. The MI fibers were then subjected to ex-vivo skin permeation studies using vertical Franz diffusion cell with Sprague Dawley male rats back skin. After 24 h, the percentage of permeated drug was 12.71 ± 0.05% for FA and 22.99 ± 0.04% for Khellin.

**Supplementary Information:**

The online version contains supplementary material available at 10.1038/s41598-025-11114-7.

## Introduction

Nanotechnology is becoming a rapidly growing field nowadays. One of the most interesting areas in nanotechnology is the synthesis and application of nanofibers^1^. They have a wide range of applications, owing to their unique properties that include elasticity, flexibility, high ultimate tensile strength and high porosity. These characteristics make nanofibers excellent candidates for use in sensors, filters^2^ and drug delivery systems^3^.

Various drug delivery systems have been investigated to increase the efficacy and decrease the toxicity while administrating a certain drug. Compared to other formulation, electrospun nanofibers became appealing for use in drug delivery as they provide great flexibility in selecting the materials to be loaded. They also offer high loading capacity, high encapsulation efficiency, cost effectiveness, ease of operation and the possibility of delivering multiple therapies simultaneously^4^. The addition of tailored recognition sites to these electrospun fibers using the molecular imprinting technology makes them even more attractive as drug delivery vehicles^5^. Molecular imprinting is a technique used to create synthetic receptors that can be described in analogy to the “lock and key model” described by Emil Fischer^6,7^. During imprinting, molecular recognition sites are introduced within the polymeric matrices making them highly selective towards the target molecules^8,9^.

Electrospinning is a versatile method that uses a high voltage to charge a pending droplet of polymer solution to draw polymer jets by electric forces, where the solvent evaporates causing the formation of fine fibers that are then collected on the nearest grounded surface as a web of nano or microfibers^10^. During electrospinning, the liquid is ejected from the tip of the needle to produce a pendant droplet resulting from the surface tension. As the droplet is electrified, the electrostatic repulsion between the surface charges of the same sign deforms the droplet into a Taylor cone, from which a charged jet is ejected. The elongating jet initially extends as a straight line, then it undergoes vigorous whipping motions and becomes an expanding helix. Thinning of the polymer jet continues until it solidifies, leading to the deposition of solid fibers on the grounded collector^10^. In general, the process of electrospinning can be divided into four main steps. The first step is charging of the liquid droplet and formation of the Taylor cone. Second, is the extension of the charged jet al.ong a straight line. This is followed by the thinning of the jet in the presence of an electric field and electrical bending instability; also called whipping instability. Lastly, the solidification and the collection of the jet as solid fibers on a grounded collector^11^.

In a previous study, electrospun scaffolds were fabricated using poly(D, L-lactide-co-glycolide) (PLGA) as a degradable matrix polymer, incorporating a functional amphiphilic macromolecule based on star-shaped poly(ethylene oxide) to enhance hydrophilicity and suppress non-specific protein adsorption. The resulting fibers enabled covalent attachment of cell-adhesion peptides, promoting specific cell-material interactions. This approach demonstrated the potential of electrospun nanofibers to serve not only as drug carriers but also as bioactive platforms capable of directing cellular behaviour, an important consideration for advanced biomedical applications^12^.

In another study, electrospun nylon nanofibers were integrated with polypyrrole-based molecularly imprinted polymers (MIPs) for the selective detection of glucose. The composite fibers exhibited high surface area, mechanical stability, and enhanced sensitivity due to the molecular imprinting technique. The system allowed for specific glucose recognition through template-specific cavities, demonstrating the applicability of electrospun MIP fibers in biosensing, highlighting the versatility of electrospinning and molecular imprinting technologies beyond drug delivery, including selective molecular recognition^13^.

Despite being a simple technique, electrospinning process involves many factors that can affect the properties of the produced fibers, increasing the complexity of the process Therefore, the application of one factor at a time (OFAT) approach for their optimization can be time consuming. The use of design of experiments (DoE) approaches such as central composite design, provides a better understanding of the electrospinning process. The central composite experimental design considers the interaction between different parameters, by examining different combinations of factors at different levels using linear regression analysis of variance (ANOVA) mathematical models which provides a clear picture of factor combination interactions^14^.

Using DoE, researchers have achieved high coefficient predictive models for fiber diameter, demonstrating precise control in fiber design, outperforming OFAT by accurately reaching targeted nanofiber dimensions^15^. Moreover, DoE based optimization has been shown to enhance drug release profiles and encapsulation efficiency, enabling tailored release kinetics in delivery systems. Such benefits cannot be obtained using conventional trial and error methods^16^. Finally, DoE facilitates scaling and reproducibility by providing statistical validation and confidence intervals, setting the foundation for robust, industrial scale electrospinning which is not feasible with OFAT methodologies^17^.

Over the past decades, natural products have played an important role in the process of drug development, either by being a major source of new drug leads, or by providing raw materials for semisynthetic drugs. Tremendous efforts have been made to isolate and identify various natural compounds and to study their effect on human body^18^.

Ferulic acid (FA) is a phenolic acid found in many vegetables and grains^19^. It is a potent antioxidant and anti-inflammatory^20^. FA is a strong free radical scavenger^21^ and it is widely used in cosmetics as a photoprotective agent, delayer of skin photoaging, and brightening component. It is mainly used as a topical antioxidant since it maintains high local concentration and low cutaneous metabolism. Additionally, FA has the ability to penetrate deeply into the skin in both neutral and acidic pH. It possesses slightly better skin penetration capacity compared to other phenolics, owing to its higher lipophilicity^22^. Despite the beneficial properties of FA, it undergoes rapid decomposition and decarboxylation^23^ which reduces its efficacy^24,25^. Researches have attempted to improve physicochemical stability of FA by its incorporation into different formulations and using controlled release technologies^26^. In one study Ouimet et al.^27^, loaded FA into poly(anhydride-ester) via solution polymerization, as a biodegradable polymer for controlled release of FA. While in another study, thermosensitive chitosan-based hydrogels were synthesized for sustained release of FA for the use in corneal wound healing^28^. Additionally, electrospinning technique was previously used to encapsulate FA in PLGA/PEO polymeric nanofibers. The free radical scavenging activity of these fibers was evaluated using di(phenyl)-(2,4,6- trinitrophenyl)iminoazanium (DPPH) assay, while MTT assay was used to determine their cytotoxicity against hepatocellular carcinoma (HepG2) cells. It was observed that the polymeric nanofibers matrix acted as protective layer for FA, enhancing its physicochemical stability and preventing its degradation throughout the whole cytotoxicity experiment. The results revealed that FA nanofibers have a promising therapeutic potential for treatment of liver cancer^29^.

Khellin is a gamma pyrone, a furanochromone derivative. It is a major constituent of *Amni visnaga.* Pure khellin exists as colourless and odourless needle-shaped crystals. It has been widely used in herbal folk medicine and was used by ancient Egyptians for treatment of many diseases^30^. For many years, khellin was used in herbal medicine for treatment of asthma, kidney^31^, psoriasis^32^ and vitiligo, which is a disease characterized by loss of pigmentation in certain area of the skin. Although the exact mechanism of action is unclear, it is believed that khellin acts by stimulating melanocytes proliferation and melanogenesis. It was found that combined khellin therapy with UVA light can effectively induce re-pigmentation in the vitiliginous areas. Khellin may be administered both systemically by oral administration and locally on the skin^31^. Topical administration of khellin helps in avoiding the adverse effects caused by oral administration in addition to enhancing drug accumulation at the desired skin site. Several formulations have been developed for topical administration of khellin such as liquid emulsions^33,34^ and semi-solids like gels and ointments^35^. Many studies were conducted to improve topical delivery of khellin using nanocarrier systems such as nanotubes^36^, nanosomes, nanovesicles, and nanostructured lipid carriers^37^.

**Fig. 1:**
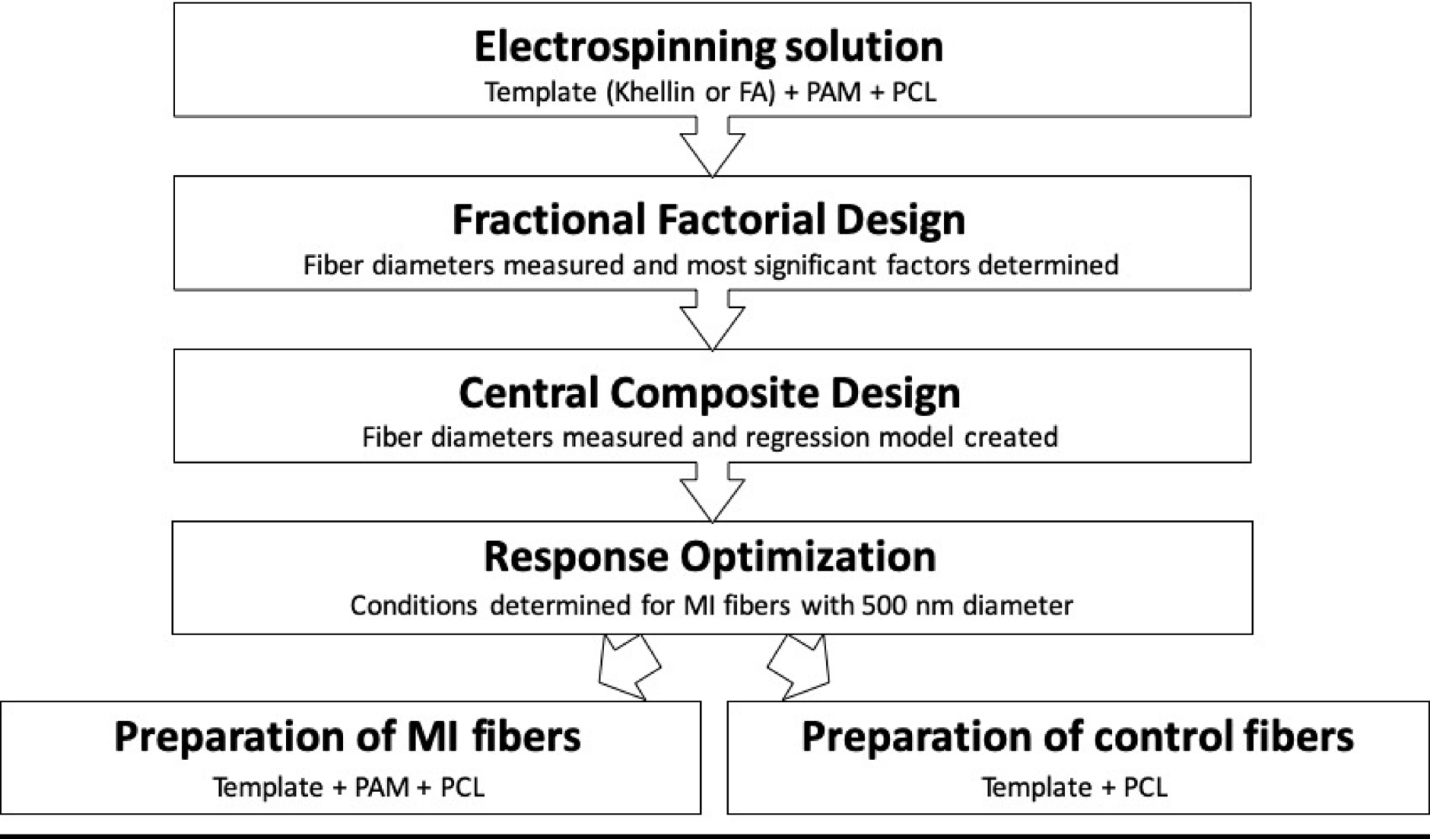
Represents flow of work for fiber production.

**Fig. 2:**
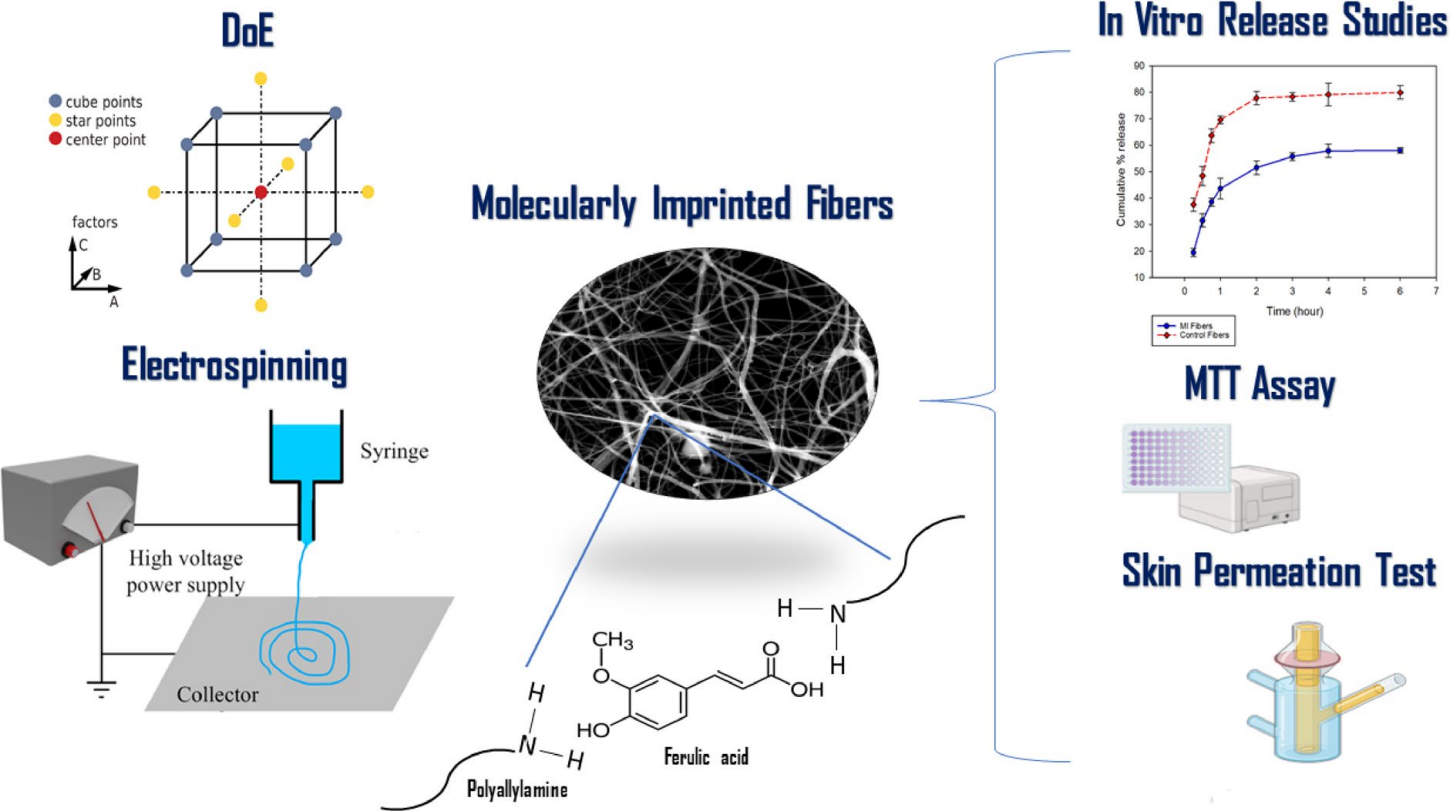
A representative figure for the major milestones in the work.

The present study aims at developing electrospun nanofibers with incorporated molecularly imprinted binding sites for two templates; ferulic acid and khellin and evaluating their potential to be used for drug delivery. Central composite design was used to study the interaction between different electrospinning parameters and predict the optimum conditions to produce electrospun fibers with an average diameter of five hundred nanometres, to be evaluated for topical drug delivery. The flow of work is represented in Fig. 1. Scanning electron microscope was used for morphology characterization of the produced fibers. This was followed by in *vitro release* studies, cytotoxicity evaluation and ex-vivo skin permeation. The major milestones in this work are represented in Fig. 2.

## Materials and methods

### Fabrication of MI fibers

The template of interest; khellin (Sigma-Aldrich, Germany) or FA (Sigma-Aldrich, Germany), was dissolved in 2.5 ml of acetone (Merck, Germany). PAM (Alfa Aesar, Germany) was dissolved in 1 ml of ultrapure water. Following which, the 2 solutions were mixed together and left to stir at room temperature for 30 min to allow the assembly between the template and PAM. High molecular weight PCL (70,000–90,000 g/mol; Sigma-Aldrich, Steinheim, Germany) was dissolved in 2.5 ml of a mixed solvent system of dichloromethane (DCM) (Sigma-Aldrich, Steinheim, Germany) and acetone with ratio (1:1v/v) and was stirred at room temperature to produce the desired concentration. This solution was then added to the previously prepared mixture and all the components were stirred for one hour at room temperature.

A vertical electrospinning setup was used for fiber fabrication. The prepared surfactant free microemulsion (SFME) was filled in a syringe with a needle connected to a high voltage supply (Leybold, Cologne, Germany). A syringe pump (CMA 402, Harvard apparatus, Holliston, MA, USA) was used to eject the SFME from the syringe. Upon application of the voltage, an electric field was conducted between the needle tip of the syringe and the collector. Fibers were produced and collected on a plastic petri dish placed on the surface of a metal collector plate.

### Design of experiments (DoE) methodology

The preparation of molecularly imprinted fibers was optimized using DoE. A fractional factorial design composed of eight runs was created for each of the templates, using Minitab^®^ 17 software (version 17.1.0, Minitab Ltd., Coventry, Warwickshire, United Kingdom); https://www.minitab.com/ to determine the most significant factors in the electrospinning process (Table 1). Pareto chart and main effects plot were obtained for the two models to determine the most significant factors affecting the morphology of the produced fibers.

**Table 1:** Fractional factorial design runs for ferulic acid and khellin.

Run order	Standard order	Template / PAM conc. (% w/v)	PCL conc. (% w/v)	Needle size (G)	Collector distance (cm)	Flow rate (uL/min)	Applied voltage (KV)
1	1	1	7	23	8	20	25
2	3	1	7	22	8	16	20
3	4	2	10	22	8	20	25
4	7	2	7	23	5	16	25
5	2	2	10	23	8	16	20
6	6	1	10	22	5	16	25
7	8	1	10	23	5	20	20
8	5	2	7	22	5	20	20

For ferulic acid, PCL concentration in addition to template and PAM concentration were the most significant factors affecting the fiber diameter. While PCL concentration was the main parameter affecting the number of beads per µm^2^. A central composite design was created using these 2 factors where default α was calculated to be 1.414. The 5 factor levels were (-1.414, -1, 0, 1, 1.414).

The design was composed of 13 runs, classified into 5 central points, 4 cube points and 4 axial points. The design factor levels for PCL concentration were (6.379, 7, 8.5, 10, 10.621%w/v), while for ferulic acid and PAM concentration they were (0.793, 1, 1.5, 2, 2.207%w/v). The 13 runs are shown in Table 2.

**Table 2:** Central composite design runs for ferulic acid/Khellin.

Run order	Standard order	Point type	PCL conc. (% w/v)	Ferulic acid and PAM conc. (% w/v)		FD (nm) ± SD	NB per µm^2^ ± SD
1	3	Cube	7	2		363 ± 199	0.001901 ± 0.0003
2	5	Axial	6.3786797	1.5		249 ± 107	0.0038718 ± 0.0006
3	2	Cube	10	1		833 ± 215	0
4	6	Axial	10.62132	1.5		1067 ± 134	0
5	10	Central	8.5	1.5		836 ± 221	0
6	13	Central	8.5	1.5		723 ± 469	0
7	7	Axial	8.5	0.7928932		657 ± 145	0
8	1	Cube	7	1		367 ± 102	0.00062 ± 0.0001
9	9	Central	8.5	1.5		790 ± 286	0
10	12	Central	8.5	1.5		735 ± 337	0.002136 ± 0.0009
11	4	Cube	10	2		1143 ± 493	0
12	11	Central	8.5	1.5		806 ± 184	0
13	8	Axial	8.5	2.2071068		821 ± 98	0

Run order	Standard order	Point type	PCL conc. (% w/v)	Khellin and PAM conc. (% w/v)	Applied voltage (KV)	FD (nm) ± SD	NB per µm^2^ ± SD
1	19	Central	8.5	1.5	22	911 ± 375	0.00638 ± 0.0002
2	14	Axial	8.5	1.5	25.364	801 ± 497	0.01517 ± 0.001
3	9	Axial	5.977	1.5	22	229 ± 66	0.02043 ± 0.0009
4	18	Central	8.5	1.5	22	945 ± 318	0
5	12	Axial	8.5	2.341	22	996 ± 406	0
6	7	Cube	7	2	24	389 ± 194	0.0618 ± 0.0006
7	10	Axial	11.023	1.5	22	1513 ± 793	0
8	16	Central	8.5	1.5	22	872 ± 98	0
9	2	Cube	10	1	20	1231 ± 103	0
10	15	Central	8.5	1.5	22	916 ± 490	0
11	1	Cube	7	1	20	460 ± 118	0.005967 ± 0.0008
12	5	Cube	7	1	24	304 ± 78	0.005302 ± 0.00005
13	8	Cube	10	2	24	1391 ± 224	0.0001434 ± 0.000001
14	4	Cube	10	2	20	1403 ± 348	0.00049976 ± 0.00003
15	11	Axial	8.5	0.6591	22	692 ± 395	0.00272 ± 0.0002
16	17	Central	8.5	1.5	22	923 ± 309	0.008344 ± 0.00009
17	3	Cube	7	2	20	394 ± 171	0.00974 ± 0.0005
18	20	Central	8.5	1.5	22	863 ± 288	0.001789 ± 0.0004
19	19	Cube	10	1	24	1374 ± 423	0
20	20	Axial	8.5	1.5	18.637	687 ± 455	0

For khellin, the most significant factors affecting the average fiber diameter were PCL concentration, template and PAM concentration and voltage. PCL concentration was the main factor affecting the number of beads per µm^2^ fibers. A central composite design was created for khellin fibers using these factors.

This design was implemented in which the axial points established new extremes for the maximum and minimum factor level of the cube specified for each factor in Minitab software. The distance between the axial and central points (alpha values) for the design is 1.682 was calculated from the equation α = n_f_^1/4^, where n_f_ is the number of points in the cube part. The 5 factor levels were (−1.682, -1, 0, 1, 1.682). The design was composed of 20 runs, they were classified into 8 cube points, 6 central points and 6 axial points. The design factor levels for PCL concentration were (5.977, 7, 8.5, 10, 11.023% w/v), for khellin and PAM concentration they were (0.659, 1, 1.5, 2, 2.341% w/v) and for the voltage they were (18.636, 20, 22, 24, 25.364 V). The central composite design runs are shown in Table 2.

The morphology of the electrospun fibers was examined using FEI Quanta 650 Environmental Scanning Electron Microscope (ESEM), USA under high vacuum at a high voltage of 10 KV with a spot size of 3.5 and working distance set to around 10 mm. The obtained images were then analysed using Image J” software (version 2.35 for Windows, 64 bit). https://imagej.net/software/fiji/downloads. The average fiber diameter for each run was obtained by performing 50 measurements on 2 different magnifications. The results were used to perform regression analysis to produce a mathematical equation describing the statistical relationship between the predictors and the responses.

The regression models were then tested by performing three different electrospinning runs for each of the two models using random combinations of the factors within the model limits. SEM images were taken and the average fiber diameter was calculated for each run. The prediction interval was then displayed for each run using Minitab at 95% confidence level.

Response optimization was then applied to determine the best combination of factors that will produce the desired fiber diameter for each of the two models. The molecularly imprinted fibers were then prepared using the optimized parameters. Control fibers for both khellin and ferulic acid were then prepared using the same condition of the molecularly imprinted fibers, but without adding PAM.

Further characterization of the fibers was done using FTIR analysis recorded in the range of 4,000–400 cm^-1^ using IRSpirit, FTIR spectrophotometer, Shimadzu (Japan).

### UHPLC method for quantification of ferulic acid and Khellin

#### Method development

ACQUITY UPLC H-Class system (Waters Corp., Milford, MA, USA) was used that consisted of Acquity UPLC Sample Manager FTN autosampler, Acquity UPLC QSM quaternary pump, ACQUITY UPLC PDA eLambda detector and Acquity UPLC CHA column manager. Aquity UPLC BEH C18 100 mm × 2.1 mm column (particle size, 1.7 μm) was used to separate analytes (Waters, Ireland). A gradient elution at a flow rate of 0.4 µL/min was conducted for chromatographic separation using 0.1% formic acid in water (A) and 0.1% formic acid in acetonitrile (B). The percentage of B started at an initial of 5% and maintained for 0.5 min, then increased up to 100% during 4 min, kept at 100% for 1.5 min, flushed back to 5% in 1 min and kept at 5% for 0.5 min. The injection volume was 10 µL and column temperature was kept at 40 °C. The analysis was conducted at wavelength of 323 nm for ferulic acid and 254 nm for khellin. System operation and data acquisition were controlled using Mass Lynx 4.1 software (Waters).

#### Method validation

The applied method was validated according to ICH guidelines in terms of linearity, LOD, LOQ and precision (inter- and intra-day). More details are provided in supplementary material.

### Encapsulated drug and In-vitro release studies

For each of the templates, 5 mg of both the molecularly imprinted and the control fibers were dissolved in 1 ml formic acid. This was followed by the addition of 4 ml of ultrapure water to precipitate PCL and 5 ml of methanol to ensure complete dissolution of the drug. The samples were then centrifuged for 45 min at 6000 rpm. The samples were analyzed using UHPLC-UV at 323 nm for ferulic acid and 254 nm for khellin. The quantification of each sample was done using a 7 point calibration curve over a concentration range of (0.01–0.4mM).

The release of both ferulic acid and khellin from the fiber matrix was determined in PBS, pH 7.40, to mimic the plasma pH and another study was done at pH 5.50 to mimic the surface of the skin^38^. For each template, 5 mg of the MIP loaded fibers and control fibers were weighed and dipped in 20 ml of PBS. The release study was performed for 6 h for ferulic acid and for 8 h for khellin in a shaker incubator maintained at 37 °C at 100 rpm. One ml aliquots were withdrawn at different time intervals and were replaced with fresh PBS. The aliquots were quantified using a 7 point calibration curve over a concentration range of (0.01–0.4mM). The cumulative amount of drug released was then calculated using Eq. 1.

1 $$Cumulative\;drug\;release\;percent = \frac{{Total\;amount\;of\;drug\;released\;from\;the\;fibers}}{{Total\:amount\:of\:drug\:encapsulated\:in\:the\:fibers\:}} \times 100$$

### MTT cytotoxicity assay

The murine melanoma B16-F10 cells purchased from American Type Culture Collection (ATCC CRL-6475™) were retrieved from *Mus musculus*, mouse. These cells were maintained as monolayers in Dulbecco’s Modified Eagle Medium (DMEM) supplemented with 10% FBS, 2mM L- Glutamine, 50 units /ml Penicillin and 50 µg/ml Streptomycin. Cells were grown in T25 or T75 flasks in humidified incubators at 37 °C, 5% CO_2_. For seeding, cells were washed with PBS and then harvested by 0.25% trypsin- EDTA (1x). Trypsin was then neutralized by the addition of the cell culture medium (containing 10% FBS). Cells were then collected by centrifugation and re-suspended in appropriate amount of cell culture medium. Finally, viable cell density was determined by direct microscopic counting on a hemocytometer using trypan blue exclusion test.

The cytotoxicity of the prepared electrospun nanofibers was assessed by MTT assay which is a colorimetric assay to detect cell viability. Mitochondrial- NAD(P)H oxidoreductases of viable cells can reduce the yellow tetrazolium salt (3-(4,5-dimethylthiazol-2-yl)-2,5-diphenyltetrazolium bromide or MTT) into purple formazan crystals^39–42^. These crystals are then solubilized by DMSO, DMF, or 20% SDS to yield an intense purple color than can be quantified using a multi-well spectrophotometer at a wave length between 500 and 600 nm. Finally, Cell viability of treated cells are compared to cell viability of untreated cells and expressed as a percentage.

The safety profile for electrospun nanofibers were assessed by MTT assay. The optimum response MI fibers of FA (8.8% PAM, 8.8% FA), Khellin (7.1% PAM, 7.1% Khellin) were chosen for testing. In addition, non-medicated control fibers (8% PAM) were tested to evaluate possible cytotoxic effects of the fiber matrix. Stocks were prepared by dispersion of the selected fibers in DMSO as a solvent. Fibers at concentrations of 100 mg/ml, 10 mg/ml and 2.5 mg/ml were solubilized by brief stirring and heating at 50 °C, a step that caused fibers to become completely soluble for not less than 30 min at room temperature. Stocks were then used to produce several treatment concentrations; noteworthy, that DMSO concentration was unified in all treatments at a concentration of 0.5%. That is, a 200x stock solution was prepared for each treatment by serial dilution from the main stocks.

On day 1, B16F10 mouse melanoma cells were seeded into 96-well plates. 200 µL of 5*10^4^ cell/ml cell suspension were added into wells. Outermost cells were not used as test wells and were only filled with PBS to avoid the edge effect.

On day 2, all stock solutions (200x) were diluted 100-folds in complete cell culture medium to yield the working solutions (2x treatment concentration). Moreover, a working solution of cell culture medium containing 1% DMSO was prepared for the untreated cells. 100 µL cell-free supernatant were removed from each well and replaced with the same volume of working solutions (2x). 4–5 technical replicates were done for each treatment.

On day 3, Cell-free supernatants were removed and 100 µL of 0.5 mg/ml MTT (in PBS) were added into each well. Cells were then incubated for 4 h to allow for enzymatic reduction of the tetrazolium salt. After that formazan crystals were dissolved by removing 50 µL from the wells, followed by addition of 150 µL DMSO. To ensure complete dissolution, well contents were mixed by pipetting, and the plates were then allowed to stand for 15 min. Plates were then scanned by Victor 1420 multi-well plate reader and absorbance readings at λ = 590 nm were recorded. Finally, the cell viability of treated wells was expressed as a percentage from the cell viability of the un-treated control, using Eq. 2.

2 $$Cell\;viability\;\% = \left( {\frac{{A_{{590}} \;treated}}{{A_{{590}} \;untreated\;cells}}} \right) \times 100$$

### Ex-vivo skin permeation studies

Sprague Dawley male rats (220–240 g) were purchased from the National Research Institute in Cairo, Egypt. The rats were housed, eight rats per cage prior to initiation of the experiment and acclimated for 7 days at 25⁰C with a controlled 12-hour light/dark cycle. All rats had free access to food pellets and water ad libitum. All animal procedures were approved by the ethics committee of the German University in Cairo (approval number PHCH-2021-05-NG) and were performed in accordance with the National Academies Guide for the Care and Use of Laboratory Animals (8th edition). All efforts were made to minimize animal distress. The rats were sacrificed by cervical dislocation and the back skin was carefully shaved using electrical clippers. The skin was separated from the subcutaneous fats and cartilages using a scalpel, washed with phosphate buffered saline and wrapped in aluminum foil and kept frozen until used.

The rat skin was cut into appropriate pieces and soaked in phosphate buffer, pH 7.4, for one hour before use. Skin permeation experiments were carried out using Franz diffusion cells, where the Skin was mounted in open two chamber Franz-type diffusion cells filled with phosphate buffer saline, pH 7.4, with the stratum corneum side up (diffusion area was 1.23 cm^2^; recipient volume 10 ml)^43^. The diffusion cells were placed in thermostatically controlled water bath resulting in a temperature of 37 °C at the skin surface. The receptor compartment was constantly stirred with a Teflon-coated magnetic bar at 200 rpm. Test formulations (10 mg of each provided sample) were then applied to the stratum corneum surface. Samples of 0.5 ml were withdrawn from the receptor medium at predetermined time intervals and replaced by fresh thermostat buffer^44^. Samples were tested for their drug content using HPLC analysis. All experiments were carried out three times, and average results were obtained.

## Results

### MI fibers preparation

Owing to its biodegradable properties, polycaprolactone was chosen as the supporting fiber matrix. Its concentration is considered one of the most important factors affecting the fiber morphology. The solvent system used for preparing the electrospinning microemulsion showed no phase separation, assuming an even distribution of PCL and PAM within the fibers. Molecular interaction between PAM and the functional groups of the templates became dominant when the solvent was completely evaporated during the electrospinning process. PCL provided additional polymer inter-chain interactions that stabilized the formed binding sites^45^. Tetrahydrofuran (THF), dichloromethane (DCM) and DCM: acetone (1:1) were examined as solvent systems for PCL. A moderately low concentration of PCL (7% w/v) was used, PAM was dissolved in water, while the template was dissolved in acetone. The electrospinning process was conducted at flow rate of 20 µl/min, applied voltage of 20 kV and collector distance of 8 cm. It was observed that when DCM: acetone (1:1) was used as the solvent system, no phase separation was observed in the solution, while the fibers were easily produced at the moderately low concentration of PCL (7% w/v).

### Design of experiments

#### Ferulic acid

*Fractional factorial design*:

A fractional factorial design was used as a screening design for ferulic acid to determine the most important factors that affect the fiber diameter and number of beads per µm^2^ during the electrospinning process. The factors screened in this step were the concentration of the template and PAM, PCL concentration, needle size, collector distance, flow rate and applied voltage. Eight runs were done and the morphologies of the produced fibers were studied using SEM and the images are shown in Figure **S1** in supplementary material.

The average diameters of the fibers and the number of beads per µm^2^ were measured using image J software and the results are shown in Table **S1**. Pareto charts of the standardized effects were used to determine the most significant factors affecting the responses (Figures S2A and S3A). On the Pareto chart, bars that cross the vertical reference line at 2.571 are statistically significant. Furthermore, normal probability plots were generated to determine the direction of the effect on the response, where negative effects are displayed on the left side of the graph while positive effects are on the right side (Figures S2B and S3B). Main effects plots were displayed to more easily visualize the effect of factors on the desired responses Figure S4.

Accordingly, PCL concentration in addition to FA and PAM concentration were chosen to further study their effects on the experimental design. The remaining factors were kept constant at the level that showed the smaller fiber diameter. The needle size was fixed at 22 G, the distance was fixed at 8 cm, the flow rate was fixed at 16 µl/min, while the applied voltage was fixed at 25 kV.

*Central composite design*:

In order to fully understand the effect of the parameters on the average fiber diameter and the number of beads per µm^2^, a central composite design was created using the 2 variables; PCL concentration in addition to FA and PAM concentration. 13 experimental runs were performed including 4 cube points (runs 1, 3 ,8, 11), 4 axial points (2, 4, 7, 13) and 5 replicates of the central point (runs 5, 6, 9, 10, 12). The morphologies of the 13 runs were analyzed using SEM (Figures S5 and S6). The average diameters number of beads per µm^2^ were determined for all the produced fibers and the results are displayed in Table 2.

Regression analysis was used to study and model the relationship between the 2 predictors and the responses. Equation 3 was generated from Minitab modelling the effect of the 2 predictors on the average fiber diameter (FD).

3 $$\begin{aligned} FD = & - 1807 + 513\;PCL\;conc. - 755\;FA,PAM\;conc. \\ & - 27.66\left( {PCLconc.} \right)^{2} + 104.7PCL\;conc. \times FA,\;PAM\;conc \\ \end{aligned}$$

The model summary shown in Table S2 reveals the p-values of the parameters considered in the model, the standard error of regression (S), the adjusted R^2^ (R-sq(adj)) and the predicted R^2^ (R-sq(pred)). Where R^2^ and adjusted R^2^were found to be 98.04% and 97.07% respectively.

Another diagnostic tool is residual plots that can be used to evaluate the goodness of the model fit. Four residual plots were obtained (Figure S7); normal probability of residuals, residuals versus fits, histogram of residuals and residuals versus order plots.

The final regression equation obtained from Minitab for average number of beads per µm^2^ (NB) is shown in Eq. 4.

4 $$NB = 0.02864 - 0.00602\:PCL\:conc.\: + 0.000315\:PCL\:conc.^{2}$$

The model has very low adjusted R^2^ (57.17%) and predictive R^2^ (30.01%); thus, it was not used for response optimization.

*Graphic modelling*:

Main effects plot was generated to visualize the effects displayed in the regression equation (Figure S8), while contour plot was used to further study the relation between the fitted response and continuous variables (Figure S9).

**Fig. 3:**
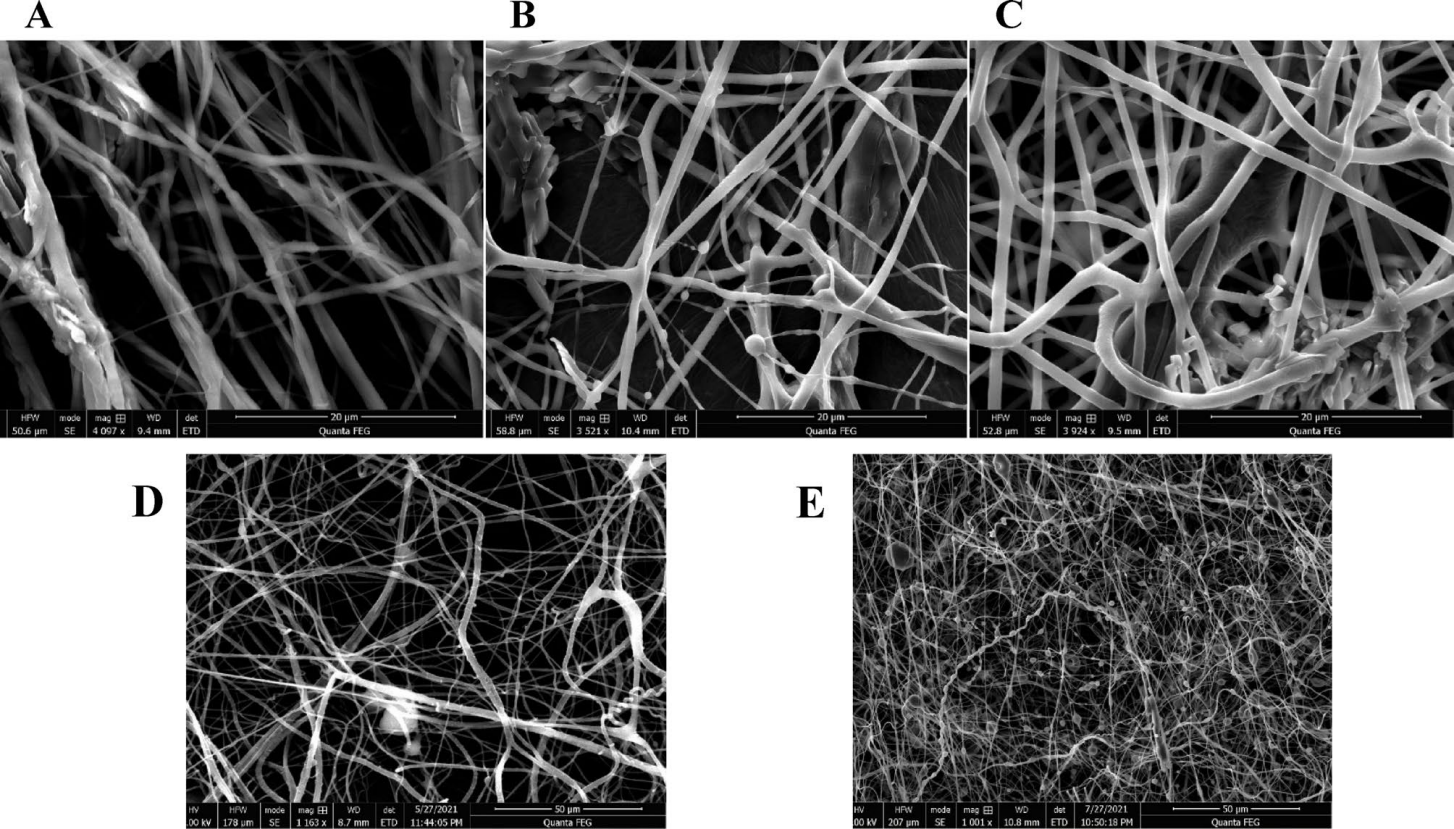
Top : SEM images of the prediction runs (**A**–**C**) for FA fibers. Bottom: SEM images of optimized FA fibers **(D)** and control fibers **(E)**.

*Testing model prediction*:

The predictability of the proposed regression model was tested by performing three electrospinning trials using a random combination of predictor values within the model limits. The predictions obtained from Minitab software were then compared to the experimental measurements generated from the electrospun fibers fabricated using the test parameters. The obtained fibers were subjected to SEM imaging (Fig. 3A, B and C) and the average fiber diameter was measured for each run. Prediction interval and predicted fit were calculated for each run using Minitab software at 95% confidence level. Table 3 shows the experimental and predicted responses for each run.

**Table 3:** Average fiber diameter results for FA /Khellin prediction runs.

	Run	PCL conc. (%w/v)	Drug/PAM conc. (% w/v)	Applied voltage (kV)	Average fiber diameter (nm)	Predicted fiber diameter	Prediction range
**FA**	1	7.5	1.2	---	547 ± 169	523.766	474.97–572.56
	2	9.5	1.2	---	889 ± 311	861.438	812.65–910.23
	3	9.5	1.8	---	1013 ± 295	1004.93	956.14–1053.72
**Khellin**	1	7.2	1.2	21	507 ± 190	464.99	379.33–532.66
	2	7.5	1.2	23	583 ± 172	564.87	502.06–627.68
	3	9.5	1.8	23	1125 ± 267	1214.97	1152.16–1277.79

**Fig. 4:**
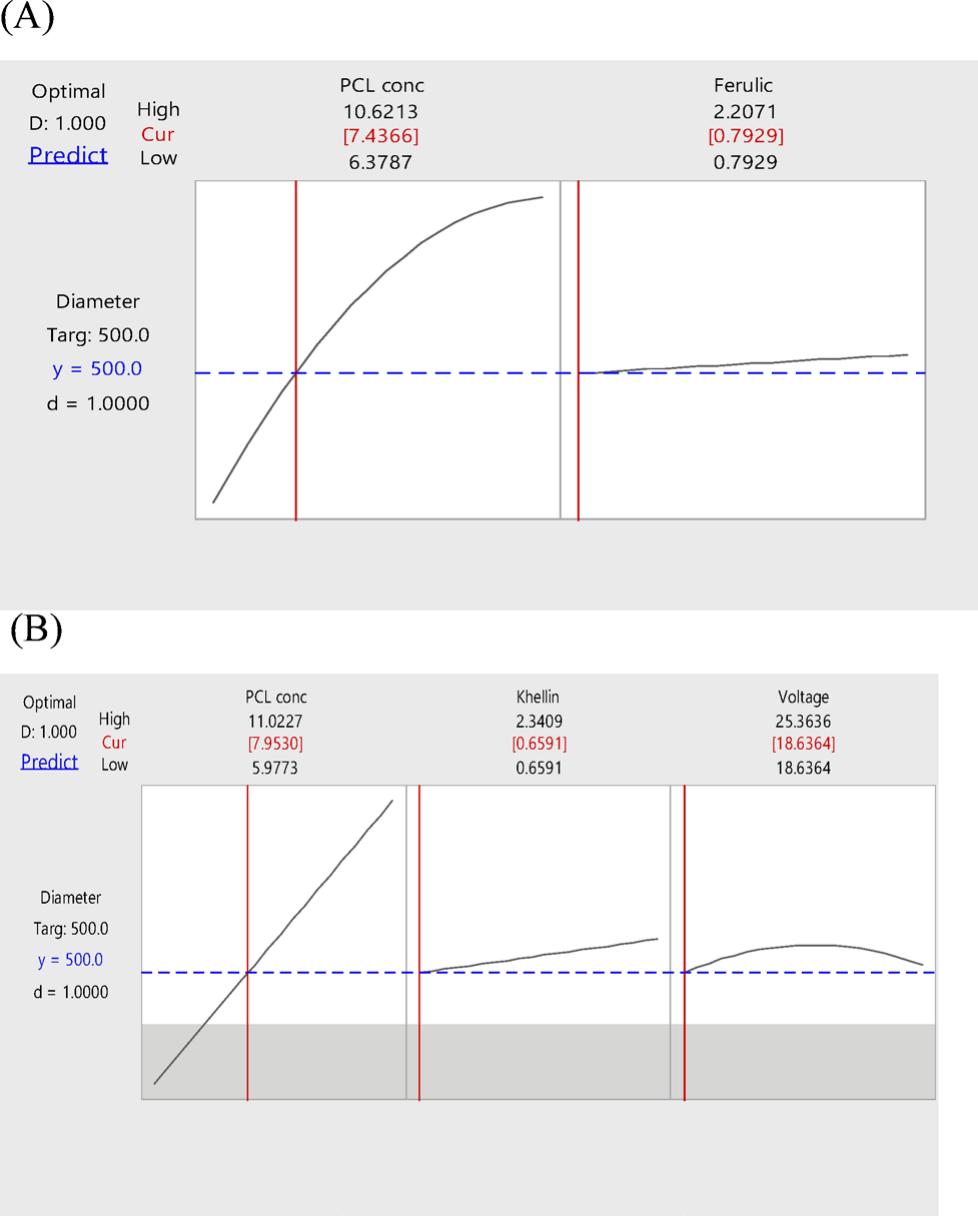
Optimization plot for FA fibers (**A**), for khellin fibers (**B**).

*Response optimization*:

Response optimizer was used to obtain the optimum conditions needed to produce fibers with an average diameter of 500 nm to be used for topical drug delivery^46^. Figure 4A shows the optimization plot, displaying the optimum conditions for producing electrospun fibers with the desired size. In order to create fibers with an average diameter of 500 nm, 7.437% (w/v) of polycaprolactone and 0.793% (w/v) of each of FA and PAM should be used.

The optimized parameters were used to prepare the solution, which was subjected to electrospinning, the fiber morphology was examined using SEM. The produced fibers showed an average diameter of 556 ± 247 nm, which lies within the prediction range of the regression model (416.9–583.1 nm). Traditional control fibers were prepared using the same procedure, without the addition of PAM. The control fibers showed smaller average diameter (351 ± 102 nm) and greater number of beads compared to the MI fibers. The morphologies of the optimized MI fibers and the control fibers are shown in Fig. 3D and E, respectively.

FTIR spectra were recorded in the range of 4,000–400 cm^− 1^ for MI fibers and control fibers, these spectra were compared to the FTIR spectrum of PCL (Figure S10 A, B and C). The spectra for MI fibers, control fibers were matching the spectrum of PCL which constitutes the main matrix of the fibers. Additional peaks were observed in the spectra of MI and control fibers at 3433 cm^− 1^ and 1519 cm^− 1^.

#### Khellin

*Fractional factorial design*:

A fractional factorial design was done for khellin to determine the most important factors affecting the fiber morphology during electrospinning. The six factors screened in this step were PCL conc., khellin/PAM conc., needle size, collector distance, applied voltage and flow rate. Eight electrospinning runs were done and the morphologies of the produced fibers were studied using SEM (Figure S11).

The average fibers diameters and the number of beads per µm^2^ were measured and the results are shown in Table S3. Pareto charts, normal probability plots, and main effects plot (Figures S12-S14) were then generated. The three most significant factors were found to be khellin and PAM concentration, PCL concentration and applied voltage and were used to create a CCD.

*Central composite design*:

A central composite design was created using the 3 factors PCL concentration, khellin/PAM concentration and applied voltage. Twenty experimental runs were performed including 8 cube points (runs 6, 9, 11, 12, 13, 14, 17, 19), 6 axial points (2, 3, 5, 7, 15, 20) and 6 replicates of the central point (1, 4, 8, 10, 16, 18). The produced fibers were subjected to SEM imaging (Figures S15- S17), while the average diameters and number of beads per µm^2^ were determined for the 20 runs and the results are displayed in Table 2.

Regression analysis was used to study and model the relationship between the 3 predictors and the response. Stepwise multiple linear regression analysis was done using the coefficients of determination (adjusted R^2^ and predicted R^2^ values) as indicators for the fitting and predictability of the model. The regression equation was obtained by step-wise elimination of the terms with the highest p-values (the terms with low significance) until no further increase in the R^2^ values was observed. A regression equation was obtained from Minitab software, displaying the effect of the 3 factors on the average fiber diameter (Eq. 5).

5 $$\:\varvec{F}\varvec{D}=\:-7283+93.5\:PCL\:conc.\:+105.3\:Khellin,\:PAM\:conc.\:+496\:Voltage-11.15\:{Voltage}^{2}$$

The model summary (Table S4) reveals the p-values of the parameters considered in the model. The model showed R^2^ value of 96.69% and adjusted R^2^ of 95.80%.

Residual plots were obtained for the model including normal probability of residuals, residuals versus fits, histogram of residuals and residuals versus orders plots (Figure S18).

The final regression model obtained from Minitab for average number of beads per µm^2^ is shown in Eq. 6.

6 $$\:\varvec{N}\varvec{B}=0.0553-0.00569\:PCL\:conc.$$

Although all terms included had p-values of less than 0.05, the model has very low adjusted R^2^ (22.13%) as well as predictive R^2^ (5.08%); thus, this model could not be used for response optimization.

*Graphic Modelling*:

The main effects plot was obtained to display the effects of different terms in the regression equation on the average fiber diameter (Figure S19).

Contour plots were obtained for different variables versus the average fiber diameter. Each plot displays a 2-dimentional view, where 2 variables are plotted on the x- and y- axes, while the other variable is kept at a constant value (Figure S20).

**Fig. 5:**
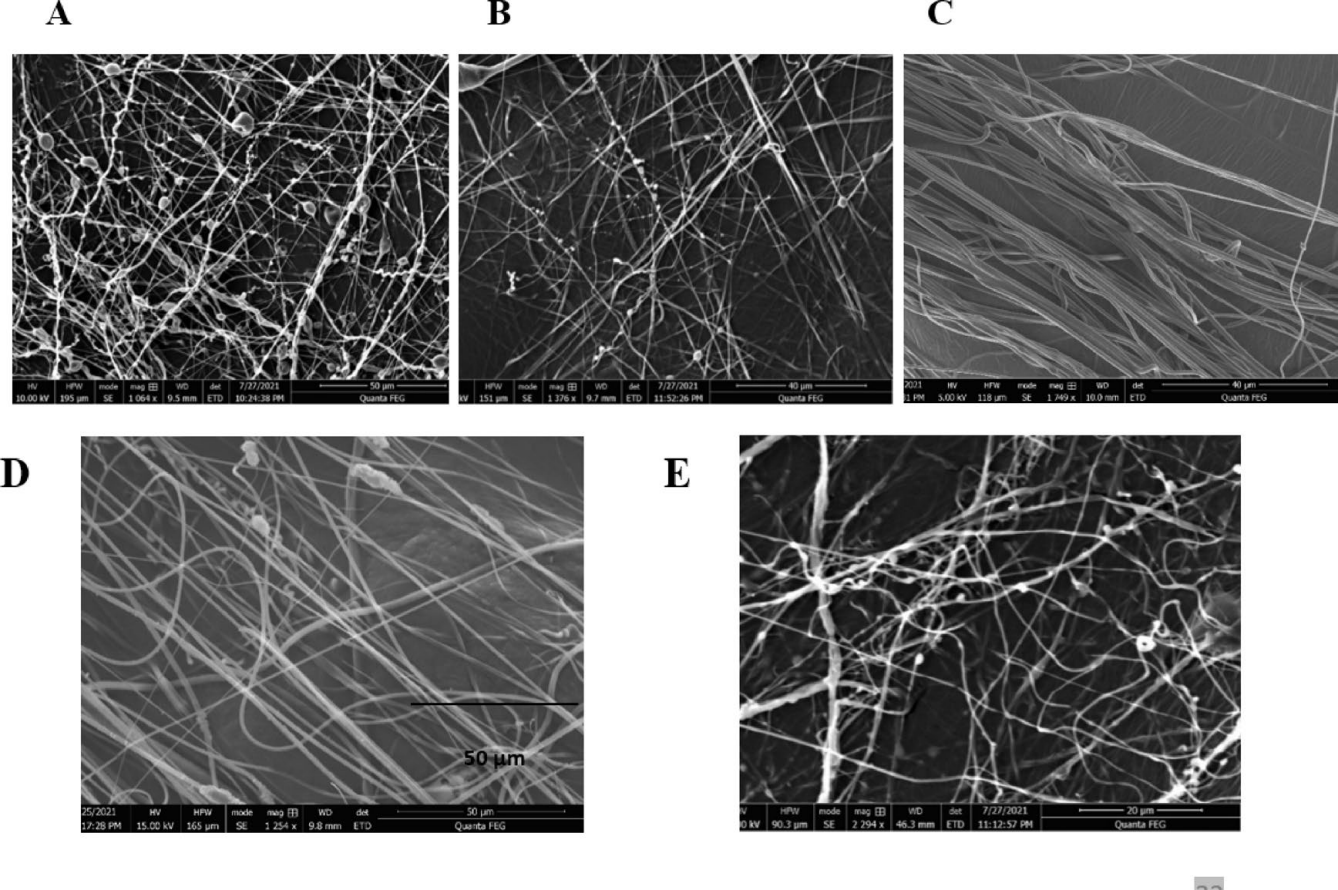
Top: SEM images of the prediction runs (**A**–**C**) for khellin fibers. Bottom: SEM images of optimized khellin fibers **(D)** and control fibers **(E)**.

*Testing model prediction*:

The predictability of the regression model was tested by performing four electrospinning runs using a random combination of the predictors within the design space. The produced fibers were subjected to SEM imaging (Fig. 5A, B and C) and the average fiber diameters were calculated. Table 3 shows the experimental and predicted responses for each run.

*Response optimization*:

The model was used to obtain the optimum parameters to be used for the production of electrospun fibers with an average diameter of 500 nm (Fig. 4B). These parameters are 7.953% (w/v) of PCL, 0.659% (w/v) of both khellin and PAM and applied voltage of 18.636 KV. The morphologies of the optimal and the control fibers are displayed in Fig. 5D and E, respectively.

FTIR spectra were recorded in the range of 4,000–400 cm^− 1^ for MI fibers and control fibers, these spectra were compared to the FTIR spectrum of PCL (Figure S10 A, D and E). The spectra for MI fibers, control fibers were matching the spectrum of PCL which constitutes the main matrix of the fibers. An Additional peak was observed in the spectra of MI and control fibers at 1651 cm^− 1^.

### Encapsulated drug and In-vitro release studies

#### Ferulic acid

The amount of encapsulated FA was determined using the calibration equation y = 191343x − 2430.4 with an R^2^ of 0.9918 obtained from a 7-point calibration curve over the concentration range of (0.01 mM – 0.4 mM).

The amount of FA encapsulated within the MI fibers was found to be 0.441 ± 0.026 mg FA/ 5 mg fibers, which is equal to 100.456% of the theoretical yield (0.439 mg FA/ 5 mg fibers). As for the control fibers, it was found that they contained 0.473 ± 0.017 mg FA/ 5 mg fibers, which is equal to 98.337% of the theoretical yield (0.481 mg FA/ 5 mg fibers).

**Fig. 6:**
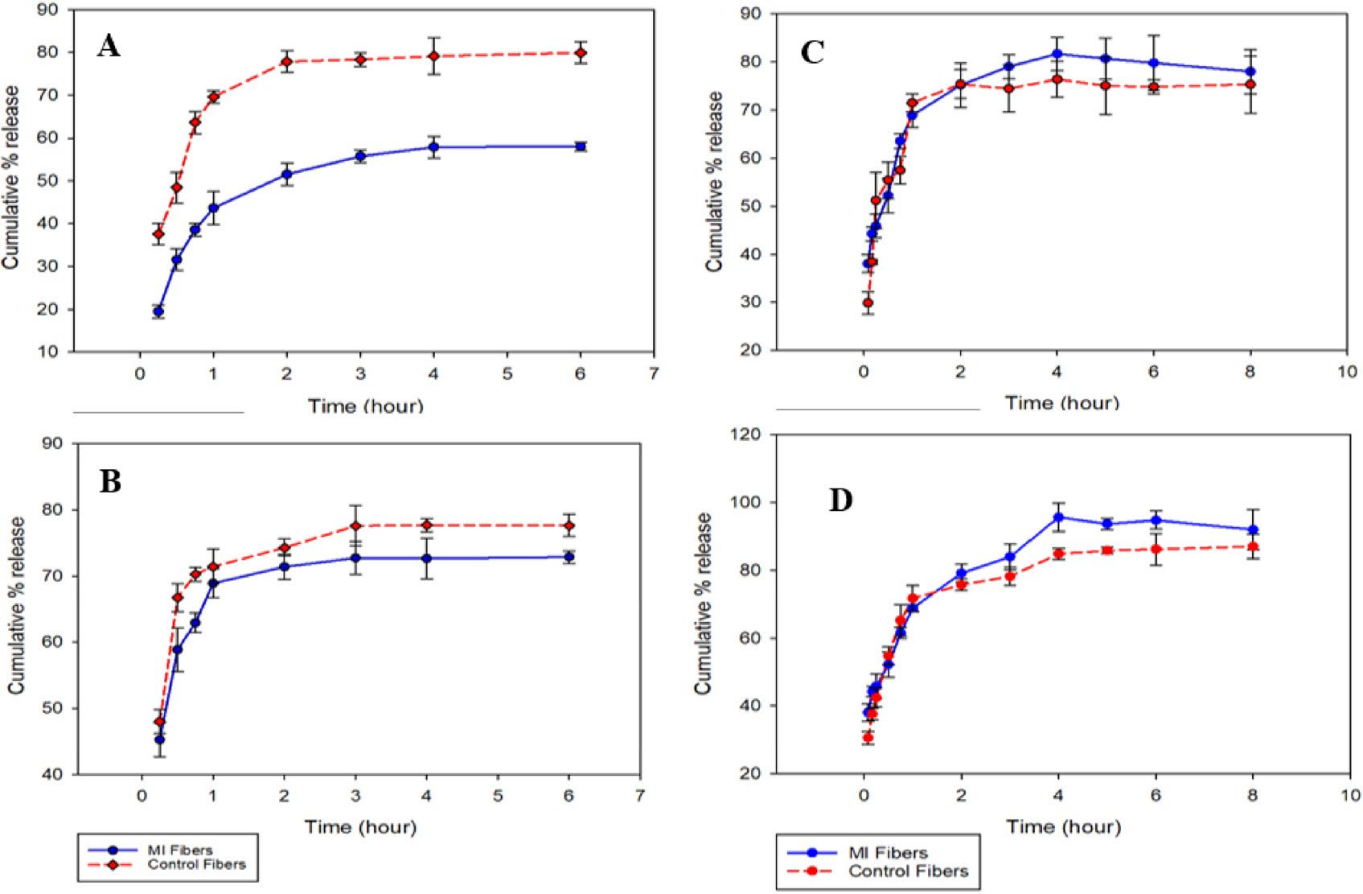
FA release from electrospun fibers in PBS with pH 7.4 (**A**) and pH 5.5, (**B**); khellin release from electrospun fibers in PBS with pH 7.4, (**C**) and pH 5.5, (**D**) at 37 °C.

The release profile of FA from the fiber matrix was determined in PBS, pH 7.40 and 5.50 for 6 h (Fig. 6A and B respectively). The amount of ferulic acid released was quantified over a seven-point calibration curve using UHPLC-PDA at wavelength 323 nm.

#### Khellin

The amount of encapsulated kellin was determined using the calibration Eq. 199161x − 524.23 with an R^2^ of 0.9985 obtained from a 7-point calibration curve over the concentration range of (0.01 mM – 0.4 mM).

The amount of khellin encapsulated within the MI fibers was found to be 0.387 ± 0.021 mg/ 5 mg fiber, which is equal to 109.014% of the theoretical yield (0.355 mg khellin/ 5 mg fibers). As for the control fibers, it was found that they contained 0.406 ± 0.013 mg khellin/ 5 mg fibers, which is equal to 106.005% of the theoretical yield (0.383 mg khellin/ 5 mg fibers).

The release of khellin from the fiber matrix was determined in PBS, pH 7.40 and pH 5.50 for 8 h (Fig. 6C and D). The amount of khellin released was quantified over a calibration curve using UHPLC-PDA at wavelength 254 nm.

### MTT cytotoxicity assay

#### Ferulic acid

The cell viability of treated B16F10 murine melanoma cells was assessed by MTT assay after treating them with solutions of the optimized FA fibers (8.8% PAM, 8.8% FA), and Non-medicated control fibers (8% PAM).

**Fig. 7:**
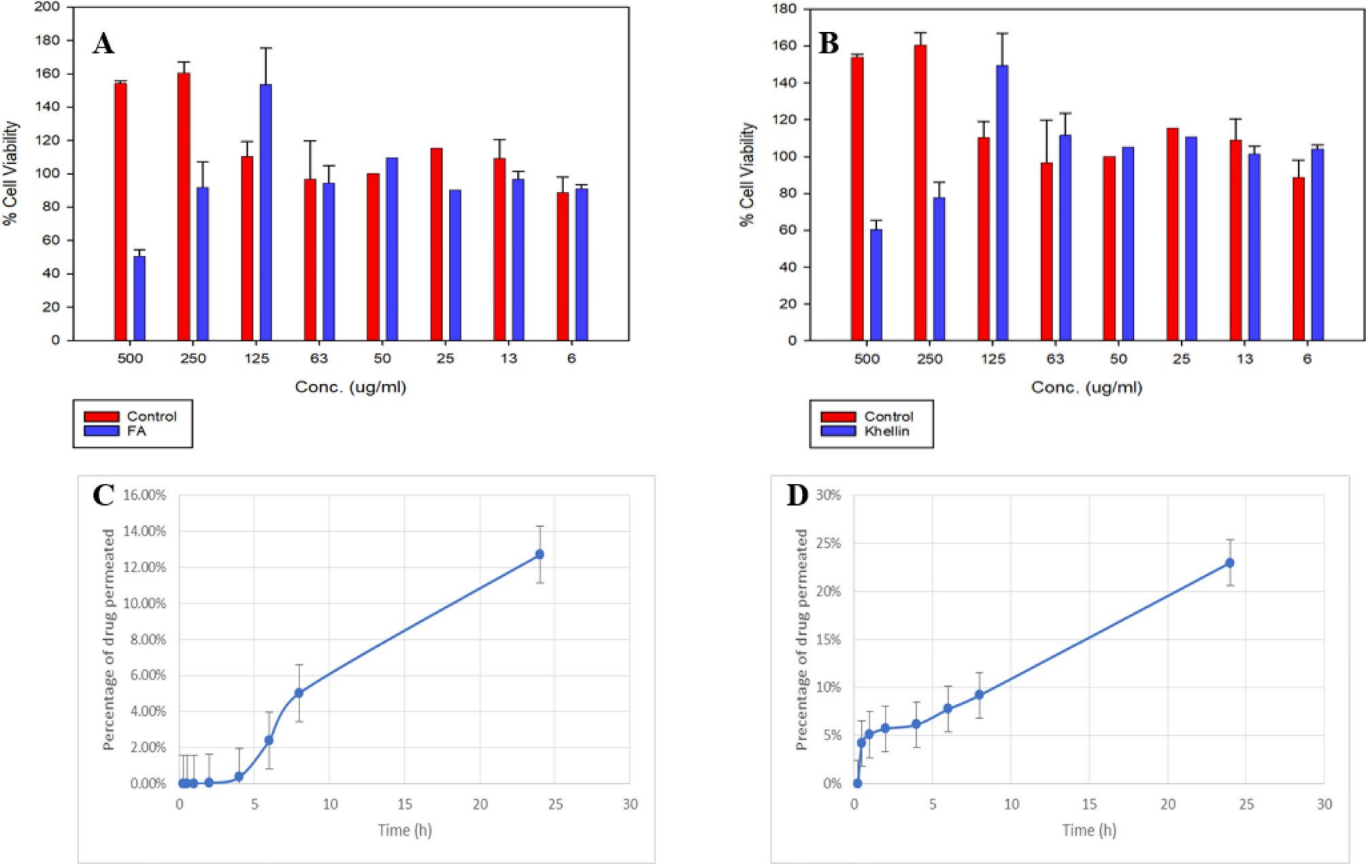
Top: Safety profile of (**A**) non-medicated control fibers vs. MI FA fibers and (**B**) non-medicated control fibers vs. MI khellin fibers. Bottom: Percent of drug permeated from **(C)** MI FA fibers and **(D)** MI khellin fibers over 24 h.

As indicated in Table S5 and Fig. 7A, the non-medicated control fibers up to 500 µg/ml were well- tolerated by the melanoma cells. On the other hand, the medicated FA fibers were also well-tolerated by the cells in concentrations up to 250 µg/ml; however, at 500 µg/ml the cells suffered remarkable cytotoxicity.

#### Khellin

The outcome of the MTT assay is indicated in Table S6 and Fig. 7B. As mentioned earlier, the non-medicated control fibers were well-tolerated by the cells up to 500 µg/ml. On the other hand, the medicated khellin fibers showed excellent safety on the cells up to 125 µg/ml, while showing 22% and 40% reduction in cell viability at 250, and 500 µg/ml fibers, respectively.

### Ex-vivo skin permeation studies

#### Ferulic acid

Ferulic acid fibers showed 12.71 ± 0.053% permeation after 24 h. The results are shown in Table S7 and Fig. 7C.

#### Khellin

After 24 h, MI khellin fibers showed 22.99 ± 0.041% permeation and the results are shown in Table S8 and Fig. 7D.

## Discussion

### Design of experiment

DoE offers clear advantages over the traditional OFAT approach in the fabrication of electrospun nanofibers for drug delivery. DoE enables simultaneous optimization of multiple parameters, demonstrating the interactions between different parameters and reducing the overall number of experimental runs^15^. In contrast, OFAT methods are inefficient, time consuming and unable to determine interaction effects, often leading to suboptimal fiber morphology and drug encapsulation^17^.

#### Ferulic acid

*Fractional factorial design*:

It has been proven that the fiber diameter can be controlled within a broad range by proper selection of the processing parameters^47^. Studying the effects of a large number of variables in an experimental design requires a lot of screening runs, which is practically and economically not feasible. Fractional factorial design is the design of choice if screening of many factors is required. This design requires only a subset of experiments that provide quite good information about the main effects and some information about the interaction effects^48,49^.

A fractional factorial design composed of eight runs was used as an initial step for screening of six different factors affecting the fiber diameter and number of beads per µm^2^ during the electrospinning process. These factors were the concentration of the template and PAM, PCL concentration, needle size, collector distance, flow rate and applied voltage. The morphologies of the fibers produced from the eight runs were studied using SEM.

The obtained results were used to create a pareto chart of the standardized effects to determine the most significant factors influencing the fiber morphology. On the pareto chart (Figure S2A), it was observed that PCL concentration in addition to FA and PAM concentration had a significant influence on fiber diameter at 0.05 level. Additionally, normal probability plot was used to determine the direction of the effect on the response. It was observed that the two factors had a positive standardized effect on the fiber diameter in which the diameter was shown to increase as each factor level increases (Figure S2B). While for number of beads per µm^2^ response, only PCL concentration was found to have a significant negative influence (Figure S3). Main effects plots were used to clearly visualize the effect of factors on the desired responses (Figure S4), which confirmed the conclusions obtained from pareto charts and normal probability plots.

PCL concentration in addition to FA and PAM concentration were then chosen to create a central composite design, while the needle size was fixed at 22 G, the distance was fixed at 8 cm, the flow rate was fixed at 16 µl/min and the voltage was fixed at 25 kV.

*Central composite design*:

The central composite design is composed of 2-level full factorial design or fractional factorial design, in addition to axial or star points and at least one central point^48^. The model is composed of three parts, a factorial design with a total of 2^f^ cube points with coordinates of x_i_ =−1 or x_i_ = + 1, an axial or star part with a total number of 2f points, with all their coordinates null except for one that is set equal to a certain value α or -α, in which α is the distance between the axial point and the central point. The third component of the design is the center point which is performed in replicates and has a coordinate of 0, referred to as n_c_. Thus, all the factors are studied on 5 levels (−α, −1, 0, 1, +α). The total number of experiments required in the CCD is calculated using the following Eq. (7)^50,51^.

7 $$\:\varvec{N}={2}^{\varvec{f}}+2\varvec{f}+{\varvec{n}}_{\varvec{c}}\:\:\left(7\right)$$

Where f is the number of factors, while n_c_ is the number of center point replicates.

Accordingly, a central composite design was created for ferulic acid fibers in order to fully understand the effect of the parameters on the average fiber diameter and the number of beads per µm^2^. The design was created using the 2 variables; PCL concentration in addition to FA and PAM concentration and 13 experimental runs were performed including 4 cube points (runs 1, 3 ,8, 11), 4 axial points (2, 4, 7, 13) and 5 replicates of the central point (runs 5, 6, 9, 10, 12).

Regression analysis was used to investigate the relationship between the predictors and the responses. Stepwise multiple linear regression analysis was done using the coefficients of determination (adjusted R^2^ and predicted R^2^ values) as indicators for the fitting and predictability of the model. The regression equation was obtained by step-wise elimination of the terms with the highest * p*-values (the terms with low significance) until no further increase in the R^2^ values was observed.

First, a regression model for average fiber diameter was created. From the model summary and regression equation, it is clear that there is a significant statistical relationship between the average fiber diameter and the linear terms of the 2 predictors in addition to the quadratic term of PCL concentration. Moreover, there was an interaction between PCL concentration and FA/PAM concentration. All the p-values were lower than 0.05 indicating that all the indicated terms in the regression equation have significant effect on the variation of the fiber diameter over the design space model.

The model was found to have both high adjusted R^2^ of 97.07% and high predicted R^2^ of 94.74%. It’s better to consider the adjusted R^2^ rather than R^2^, as the adjusted R^2^ accounts for the number of predictors added in the model. The predicted R^2^ indicated how well a regression model predicts the response when different conditions are applied. The model also revealed a low standard error of regression (S) of 44.9466. S represents the average distance by which the observed values fall from the regression line. Smaller values indicate that the observations lie closer to the fitted line. Moreover, four residual plots were obtained; normal probability of residuals, residuals versus fits, histogram of residuals and residuals versus order plots (Figure S7). The results were added in supplementary material.

As for the number of beads regression model, although all terms included had p-values of less than 0.05, the model has very low adjusted R^2^ (57.17%) as well as predictive R^2^ (30.01%); thus, this model does not possess a good predictive ability to be used for response optimization. However, it was observed that PCL concentration of 7% (w/v) or less yielded a large number of beads, while increasing the PCL concentration above 7% resulted in very few or no beads. Moreover, it was previously reported that the polymer concentration has the most significant influence on beads formation, where increasing the polymers concentration causes decrease in the number of beads^14^.

*Graphic modelling*:

To avoid any error that might be introduced into the current study due to changes in the ambient conditions, all the experimental runs were performed in one day, with ambient temperature of around 25 °C and intermediate humidity. Higher temperatures were avoided, as they might cause a decrease in the viscosity of the prepared emulsion, leading to smaller fiber diameters. Days with very low humidity were avoided as this might cause fast solvent evaporation and drying leading to needle clogging^52^.

To show the effects displayed in the regression equation, main effects plot was generated (Figure S8). Main effects plot shows the fitted response means for each factor in the regression equation, while keeping the other variables constant. Thus, displaying the relation between each factor and the response in isolation from the other factors.

The molecular weight of the used polymer has a great impact on the solution viscosity as it is responsible for the number of polymer chain enlargements in the solution^53^. High molecular weight polycaprolactone (70,000–90,000 g/mol) was used in this model.

The polymer concentrations used in this study were within the range (6.38–10.62%). Thinner diameters were observed upon using low polymer concentrations which agrees with what is previously reported in literature. This might be attributed to higher mobility of the polymer chains and stronger instabilities of the jets during electrospinning, which induces higher stretching of the polymer jet and may lead to the formation of beads. As the polymer concentration increased, larger fibers with lower number of beads were produced. This is probably due to the increase in the solution viscosity which increases the enlargements of the polymer chains in the solution, limiting the jet ability to stretch, leading to the production of larger and more uniform fibers^54^.

Increasing the concentration of any of the solutes (especially PAM), causes an increase in the solution viscosity. Accordingly, this causes an increase in the produced fiber diameter. Therefore, there was a slight increase in the fiber diameter observed upon using higher concentrations of FA and PAM.

Contour plots graphically represent the relation between the fitted response and two continuous variables. It displays a 2-dimensional view, where the two variables are plotted on the x- and y- axes. The points having the same response are connected to produce contour lines of constant responses. Figure S9 displays the contour plot of PCL concentration displayed on the x-axis and FA/PAM concentration on the y-axis. The darkest blue region represents the lowest average diameter values, while the darkest green region represents the highest values. The plot reveals that the average fiber diameter is mainly responsive to change in PCL concentration.

*Testing model prediction*:

The predictability of the proposed regression model was tested by performing three electrospinning trials using a random combination of predictor values within the model limits. The predictions obtained from Minitab software were then compared to the experimental measurements generated from the electrospun fibers fabricated using the test parameters. The obtained fibers were subjected to SEM imaging and the average fiber diameter was measured for each run.

Prediction interval and predicted fit were calculated for each run using Minitab software at 95% confidence level. All the experimental results were found to lie within their predicted ranges. This concludes that the regression model has good predictive ability and can be used to optimize the production of FA fibers with specific diameters.

*Response optimization*:

In order to obtain the optimum conditions for production of fibers with 500 nm diameter, response optimizer was used. Figure 4A shows the optimization plot, where these conditions were found to be 7.437% (w/v) of polycaprolactone and 0.793% (w/v) of each of FA and PAM.

The optimized parameters were used to prepare the solution which was subjected to electrospinning, the fiber morphology was examined using SEM. The produced fibers showed an average diameter of 556 ± 247 nm, which lies within the prediction range of the regression model (416.9–583.1 nm). Traditional control fibers were prepared using the same procedure, without the addition of PAM. The control fibers showed smaller average diameter (351 ± 102 nm) and greater number of beads compared to the MI fibers. This might be due to the absence of PAM which resulted in a solution with lower viscosity.

The results of FTIR analysis of MI fibers and control fibers revealed that the spectra are identical, the main vibrational peaks in both spectra are attributed to PCL as observed in Figure S10 (A, B and C). This is expected as PCL constitutes the main matrix of the fibers. There were no differences observed between the spectrum of MI fibers; Figure S10 B and control fibers; Figure S10 C. Two additional peaks were observed in the spectra of MI fibers and control fibers and are absent in the PCL spectrum. The additional peak at 3433 cm^− 1^ could be mainly attributed to the OH stretching vibration of FA while the peak at 1519 cm^− 1^ could be assigned to the aromatic C = C stretching of FA.

#### Khellin

*Fractional factorial design*:

A fractional factorial design was created for khellin using six factors; PCL conc., khellin/PAM conc., needle size, collector distance, applied voltage and flow rate. Eight electrospinning runs were done and the morphologies of the produced fibers were studied using SEM, pareto charts, normal probability plots, and main effects plot were then generated. Factors that had a strong influence on the fiber diameter were found to be PCL concentration, khellin/PAM concentration and applied voltage (Figure S12A); the first two factors had a positive effect on the response, while the latter had a negative effect (Figure S13A). For the number of beads per µm^2^ response only PCL concentration had a significant negative effect (Figures S12B & S13B). The main effects plots displayed in Figure S14, also showed that PCL concentration had the biggest influence on fiber diameter, followed by Khellin/PAM concentration and applied voltage. As for the number of beads, PCL concentration appeared to have the most significant influence, while the rest of the factors has almost equal effects. The 3 aforementioned parameters were then used to create a central composite design composed of 20 runs.

*Central composite design*:

A central composite design was created for khellin using 3 factors; PCL concentration, khellin/PAM concentration and applied voltage. The design was composed of 20 runs including 8 cube points, 6 axial points and 6 replicates of the central point. Afterwards, regression analysis was used to model the relation between the predictors and the responses.

First, a regression equation was created to display the effect between the aforementioned factors and average fiber diameter (Eq. 5). The model summary (Table S4) reveals the p-values of the parameters considered in the model, the standard error of regression (S), the adjusted R^2^ and the predicted R^2^. It is clear that there is a significant statistical relationship between the fiber diameter and the linear terms of PCL concentration, Khellin/PAM concentration, applied voltage and the quadratic term of the applied voltage. Moreover, there is no significant interaction between any of the model terms. All the p-values were lower than 0.05 indicating that all the indicated terms in the regression equation have significant effect on the fiber diameter. The model was found to have both high adjusted R^2^ of 95.80% and a high predicted R^2^ of 93.23%. It also revealed a low standard error of regression (S) of 78.7654.

Residual plots were obtained for the model including normal probability of residuals, residuals versus fits, histogram of residuals and residuals versus orders plots (Figure S18). Results were added in supplementary material.

As for the regression model for average number of beads, the model showed very low adjusted and predictive R^2^ and was not used for response optimization.

*Graphic modelling*:

Main effects plot displayed in Figure S19, shows the effect of different factors on the fiber diameter.

High molecular weight polycaprolactone (70,000–90,000 g/mol) was used in this model over a concentration range of (5.98–11.02% w/v). The produced fiber diameter is mainly affected by the polymer concentration. Lower PCL concentrations resulted in the formation of thinner fibers, while higher concentrations resulted in the formation of thicker fibers. Increasing the concentration of khellin and PAM also caused an increase in the average fiber diameter. However, the effect was less pronounced than the PCL effect. The effect of the applied voltage was studied over the range of 18.6–25.4 kV. The curvilinear impact of the applied voltage is shown in Figure S19. Within the studied range in the model, the increase in the applied voltage induced the ejection of more fluid in the jet causing the production of fibers with larger diameters. However, increasing the voltage over 22 kV caused a reduction in the fiber diameter. This could be attributed to the increase in the electrostatic repulsive force on the fluid jet, causing more solution stretching^53^.

Figure S20 shows the contour plots for different variables versus the average fiber diameter. The results reveal that the fiber diameter is highly affected by the change in PCL concentration.

*Testing model prediction*:

Four electrospinning runs were conducted to test the predictability of the regression model. Prediction interval and predicted fit were calculated for each run at 95% confidence level. Table 3 shows the experimental and predicted responses for each run. All the experimental results were found to lie within the prediction ranges. This suggests that the model has good predictive ability and can be used for the optimization of khellin electrospun fibers with specific diameters.

*Response optimization*:

The regression model was used ns for production of desired fibers with an average diameter of 500 nm. The produced fibers showed an average diameter of 517 ± 122 nm which lies within the prediction range of the model (386.7–613.3 nm). The same conditions were used to produce control fibers by directly loading khellin within the PCL matrix without adding PAM. The control fibers showed an average diameter of 386 ± 178 nm.

The results of FTIR analysis of MI fibers and control fibers revealed that the spectra are identical, the main vibrational peaks in both spectra are attributed to PCL as observed in Figure S10 (A, D and E). This is expected as PCL constitutes the main matrix of the fibers. There were no differences observed between the spectrum of MI fibers; Figure S10 D and control fibers; Figure S10 E. An additional peak was observed in the spectra of MI fibers and control fibers and was absent in the PCL spectrum. This additional peak at 1651 cm^− 1^ could be mainly attributed to C=O stretching vibration of khellin.

### Encapsulated drug and In-vitro release studies

#### Ferulic acid

The amount of encapsulated FA was determined. For the MI fibers, it was found to be 0.441 ± 0.026 mg FA/ 5 mg fibers, which is equal to 100.456% of the theoretical yield (0.439 mg FA/ 5 mg fibers), while the control fibers, contained 0.473 ± 0.017 mg FA/ 5 mg fibers, which is equal to 98.337% of the theoretical yield (0.481 mg FA/ 5 mg fibers).

In-vitro release studies were conducted in PBS at pH 7.4 and pH 5.5 to study the release of FA from the electrospun fibers. Figure 6A represents the release of ferulic acid in PBS, pH 7.4. The MI fibers showed slower burst release than the traditional control fibers. The initial burst release of the control fibers after 15 min of the release study was 37 ± 2.5%, while the MI fibers released only 19.4 ± 1.5% of FA at the same time of the study. After 6 h, the cumulative amount of FA released from the MI fibers was 58.1 ± 0.95%, while the control fibers released 79.9 ± 2.5%. In addition to their larger diameter, the imprinted fibers provided extra specific hydrogen bonds formed between FA and the functional polymer PAM. This suggests that MI fibers can be used for sustained release of FA.

Figure 6B shows the release of ferulic acid in PBS, pH 5.5. Both the imprinted and non-imprinted fibers showed a comparable release pattern. The initial burst release of the control fibers was 48 ± 1.8% which is only slightly higher than the release of the MI fibers (45 ± 2.6%). After 6 h, the cumulative % release was 72.9 ± 0.9% for the MI fibers and 77.6 ± 1.6% for the control fibers. This might be due to the acidic pH which disrupted the hydrogen bonding between the PAM and FA, leading to faster release of FA from the imprinted fibers (compared to the neutral pH).

#### Khellin

The encapsulated khellin within the MI fibers was found to be 0.387 ± 0.021 mg/ 5 mg fiber, which is equal to 109.014% of the theoretical yield which was calculated as 0.355 mg khellin/ 5 mg fibers. While the control fibers contained 0.406 ± 0.013 mg khellin/ 5 mg fibers; 106.005% of the theoretical yield (0.383 mg khellin/ 5 mg fibers).

In-vitro release studies were conducted for khellin fibers using similar conditions as FA fibers. There was no significant difference between the release of khellin from both the MI and the control fibers in either of the 2 pH values used (Fig. 6C and D). This suggests that the imprinting process for khellin was not successful under these conditions. This might be due to the low solubility of khellin in water which did not allow proper interaction between khellin and PAM (which is mainly soluble in water)^55^.

### Cytotoxicity

#### Ferulic acid

The MTT results shown in Fig. 7A and Table S5 show that the non-medicated control fibers were well- tolerated by the melanoma cells for up to 500 µg/ml. While the medicated FA fibers were well-tolerated by the cells in concentrations up to 250 µg/ml; however, at 500 µg/ml remarkable cytotoxicity was observed. The difference between the effect of the non-medicated fibers and the FA fibers at this concentration could be attributed to the anticancer activity of FA. The estimated FA content at 500 µg/ml is around 44 µg/ml (226.5 µM).

It was previously reported that FA exhibits anticancer activity at lower concentrations against breast cancer MDA-MB-231 cell line. FA demonstrated significant antitumor and anti-metastatic activity in breast cancer cells by modulating the epithelial-to-mesenchymal transition (EMT) process. Treatment with FA suppressed cellular migration and invasion while enhancing the expression of epithelial markers and downregulating mesenchymal markers, indicating a reversal of EMT. These molecular changes were associated with decreased tumor aggressiveness, suggesting potential use of FA in inhibiting metastasis and progression of breast cancer^56^. Additionally, it was also effective against 143B and MG63 Osteosarcoma cancer cell lines. In this study, ferulic acid (FA) was found to inhibit proliferation and induce apoptosis in osteosarcoma cells by targeting the PI3K/Akt signaling pathway. FA treatment led to a marked reduction in cell viability and colony formation, accompanied by increased apoptotic markers. The suppression of the PI3K/Akt axis appeared to be a key mechanism through which FA exerted its anticancer effects, highlighting its therapeutic potential in osteosarcoma management^57^.

In another study the anticancer effects of FA were examined on several melanoma cell lines, including B16F10. FA treatment (with IC₅₀ values in the low micromolar range) significantly reduced cell viability and colony formation with suppression of FGFR1activated PI3KAkt signaling. In vivo, FA inhibited melanoma growth and angiogenesis, demonstrating potent anti-melanoma activity^58^.

In conclusion, FA medicated fibers seem to be generally safe and biocompatible. In the future, we will be interested to test the hypothesis that these fibers could be utilized in bigger amounts to treat skin cancers.

#### Khellin

As mentioned earlier, the non-medicated control fibers were well-tolerated by the cells up to 500 µg/ml, while the medicated khellin fibers were safe for up to 125 µg/ml. However, the medicated fibers showed 22% and 40% reduction in cell viability at 250, and 500 µg/ml fibers, respectively (Fig. 7B and Table S6).

The remarkable difference in cell viability between the control non-medicated fibers and the khellin loaded fibers at these concentrations indicate that the anticancer activity of khellin was the major contributor to the drop-in cell viability. The estimated khellin concentrations at 250, and 500 µg/ml of fibers are 35.5 µg/ml (136.4 µM) and 17.75 µg/ml (68.2 µM), respectively. Additionally, khellin at these concentrations was reported to have anti-tumor activity against HeLa, HCT-116 m HEPG-2 and MCF-7 cancer cell lines^59^.

In a previous study, [^131^I]-labelled khellin preferentially localized in tumour tissue in vivo in a murine model, with molecular docking suggesting PI3K and VEGFR as key targets. This indicates selective tumour affinity and possible therapeutic or diagnostic applications, supporting its biological activity in vivo^60^.

These results suggest that the selected khellin medicated fibers are safe to use topically, while having the potential to be used in treatment of melanoma at higher concentrations, which is certainly something we are interested in testing in the near future.

### Ex-vivo skin permeation studies

#### Ferulic acid

Ex-vivo skin permeation studies were conducted using Sprague Dawley male rats back skin to evaluate the potential of the fabricated fibers to be used as transdermal drug delivery system for FA. The medicated fibers showed 0% permeation of the drug for the first hour, after which FA started to slowly permeate the skin showing 0.05% permeation after 2 h of the study. Afterwards, the permeation of FA started to increase gradually to reach 5.03 ± 0.030% after 8 h. By the end of the experiment; after 24 h, only 12.71 ± 0.053% of FA was permeate(Fig. 7C). The permeation experiment showed much slower release of FA compared to in-vitro release studies because the drug needs longer time to penetrate through skin barrier. This verifies the importance of ex-vivo studies to obtain more accurate results about release pattern of the drug and its permeation through skin layers^61^. The results show that FA fibers showed slower and more sustained release pattern compared to previously reported FA acid formulations, such as different FA nano-emulsion based gels that showed rat skin permeation ranging from 60 to 97% after a 24 h study conducted by Harwansh et al.^62^. This suggests that the fabricated fibers could be used as sustained release delivery system for FA.

#### Khellin

The permeation of khellin through rat skin reached 4 ± 0.021% after 30 min of the study. The cumulative amount permeated increased gradually to reach 23 ± 0.041% after 24 h (Fig. 7D). It is observed that the permeation of khellin through the skin is much faster compared to FA. This pattern can be explained by the fact that imprinting of khellin using PAM was unsuccessful as previously confirmed by the in-vitro release studies in PBS. The lack of interaction between khellin and PAM may cause faster release of the drug from the fibers. Additionally, the hydrophobic nature of khellin molecule^63^ could enhance its penetration and permeation through the skin, compared to FA. However, the fabricated khellin fibers showed slower permeation rate compared to other reported khellin topical formulations such as such as hydroxyethyl cellulose hydrogel reported by Risaliti et al.^64^ that showed 42% permeation after 24 h in a similar experiment. These results reveal that even though khellin imprinting was unsuccessful, the entrapment of khellin within PCL fibers, slows the release of khellin when applied to skin, suggesting that these fibers could be used as topical formulations for sustained release of khellin.

## Conclusion

This study proposed the use of biocompatible electrospun molecularly imprinted nanofibers for drug delivery of khellin and ferulic acid. The molecular recognition sites have been added to the polycaprolactone matrix using polyallylamine as a functional polymer. Design of Experiment approach was used to determine the effect of different electrospinning parameters on the morphology of the fabricated fibers. The produced fibers were examined using SEM. PCL concentration was found to be the most significant factor affecting the fiber diameter and the number of beads per µm^2^ in both ferulic acid model and khellin model. Different prediction runs were performed using a random combination of parameters within the design space to test the ability of the regression models to predict the fiber diameter upon using different electrospinning parameters. For ferulic acid model, the optimum conditions to produce fibers with an average diameter of 500 nm were found to be PCL concentration of 7.437% (w/v) in addition to template and PAM concentration of 0.793% (w/v). For khellin model, the optimum conditions were an applied voltage of 18.6 kV, PCL concentration of 7.953% (w/v) in addition to template and PAM concentration of 0.659% (w/v). For each template, in vitro release studies were done in PBS of pH 7.40 and pH 5.50. The release profiles of the imprinted fibers were compared to traditionally fabricated control fibers. For ferulic acid, the results showed slower burst release compared to the control fibers in neutral pH (7.40). However, there was no significant difference between the imprinted and the control fibers at pH 5.5. For khellin, both the imprinted and the control fibers showed comparable release profiles in both of the used pH values, suggesting that the process of the imprinting was not successfully achieved and needs further modification. MTT assay was conducted to evaluate the cytotoxicity of both FA and khellin fibers against murine melanoma B16F10 cell lines. FA medicated fibers were well-tolerated by the cells in concentrations ranging from 6 µg/ml up to 250 µg/ml. However, using 500 µg/ml of the fibers caused significant cytotoxic effect. The observed cytotoxicity at this concentration is probably attributed to FA which has reported anticancer effects at similar concentrations. As for khellin fibers, no cytotoxicity was observed up to a concentration of 125 µg/ml, while 250 µg/ml. and 500 µg/ml of the fibers showed significant reduction in the cell viability. This also suggests that the observed cytotoxicity is due to the potential anti-tumour activity of khellin at these concentrations. Both FA and khellin fibers were then subjected to ex-vivo skin permeation studies on Sprague Dawley male rats back skin. After 24 h, the percentage of permeated drug was 12.71 ± 0.053% for FA and 23 ± 0.041% for khellin. These results suggest that PCL/PAM fibers could be safely used for delivery of tolerated concentrations of FA or khellin. Also, the potential use of these medicated fibers for treatment of different types of cancer should be further investigated.

## Electronic supplementary material

Below is the link to the electronic supplementary material.

Supplementary Material 1

## Data Availability

The data supporting the findings of this research are available within the article and its supplementary information.
