# Supplementary material for: Design, synthesis and medical prospects of electrospun molecularly imprinted fibers
Source: Sci Rep. 2025 Jul 18;15:26082. doi: 10.1038/s41598-025-11114-7 (PMC12274513; doi:10.1038/s41598-025-11114-7)
Supplement: Supplementary file 1 — Supplementary Material 1 [file 41598_2025_11114_MOESM1_ESM.docx]

Design, Synthesis and Medical Prospects of Electrospun Molecularly

Imprinted Fibers

Sarah H. Megahed^1^, Mohammad Abdel-Halim^1^, Yahia I. El-shabrawy^2^, Engy M. Saad^1^, Amr Hefnawy^3^, Heba Handoussa^4^, Boris Mizaikoff^5,6^_,_ Nesrine A. El Gohary^1,*^


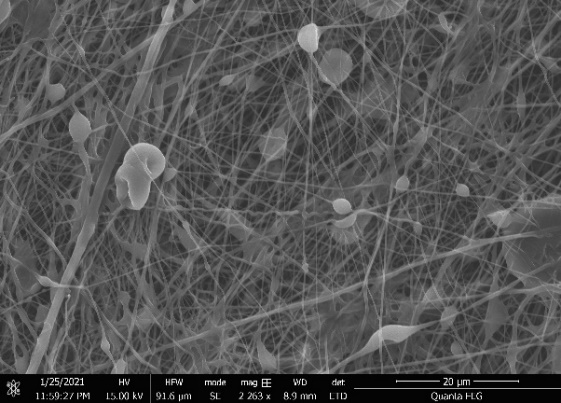

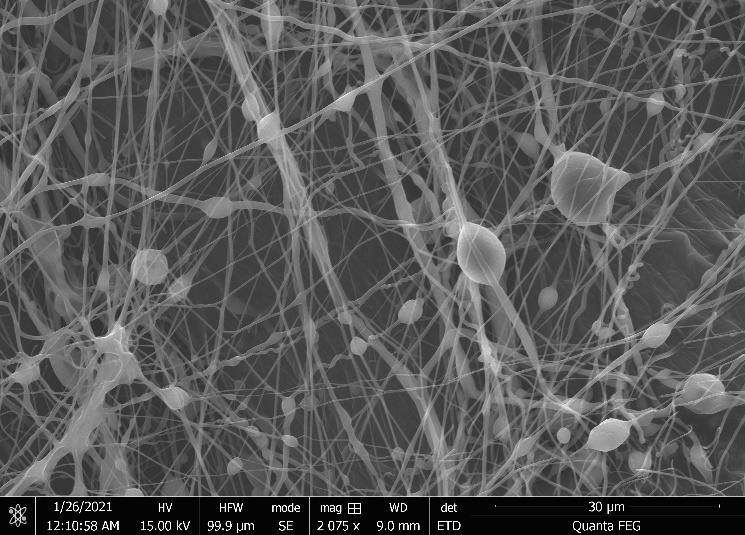

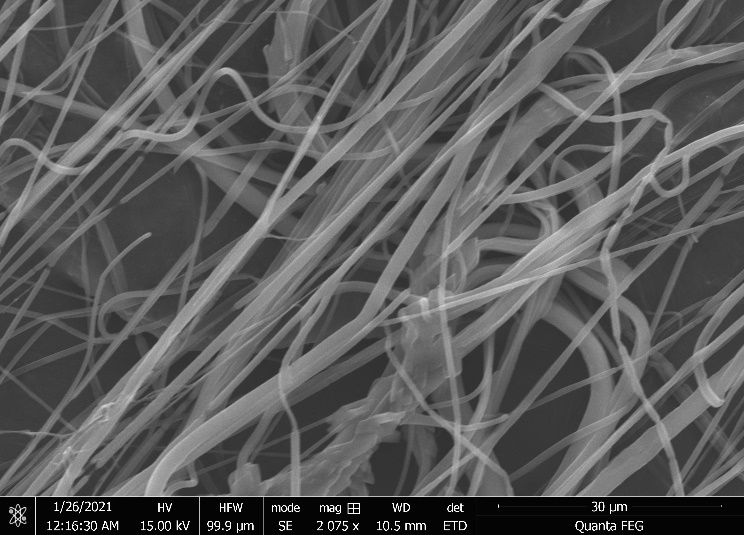


**1**

**2**

**3**

**4**


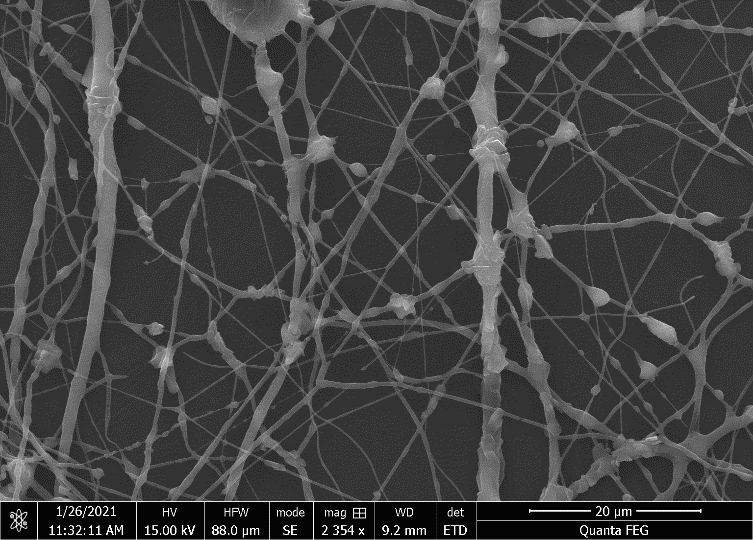

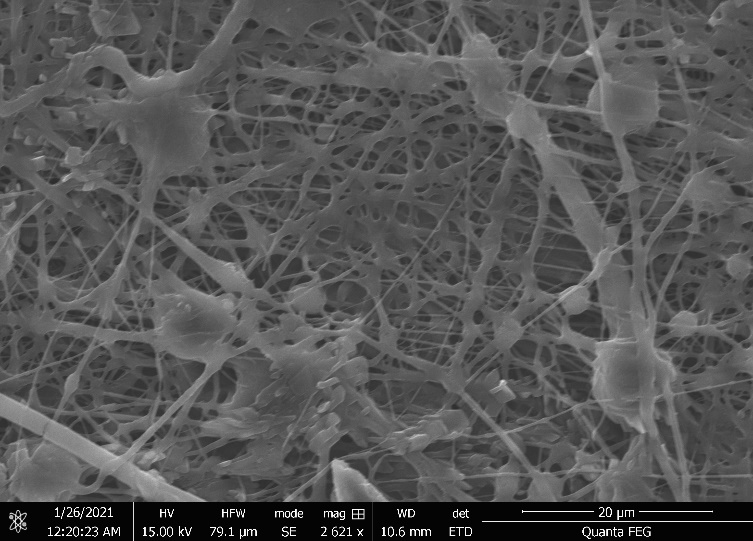

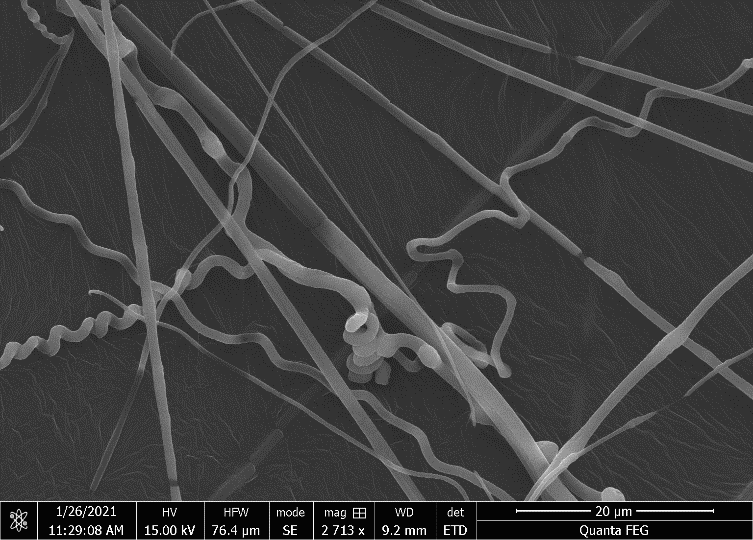


**6**

**5**


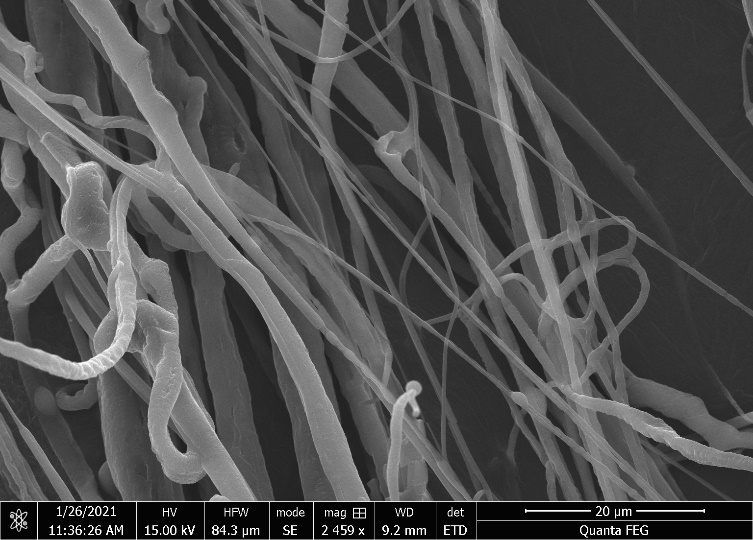


**7**

**8**


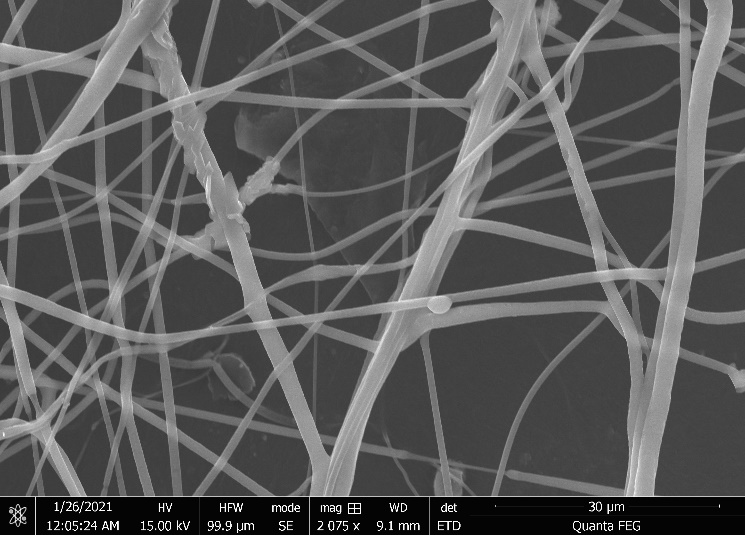


**Figure S1:** SEM images (trials 1-8) of the fractional factorial design for FA.

**A**

**B**

**Figure S2:** Pareto chart of standardized effects (A) and normal probability plot (B) for average fiber diameter for FA.

**Figure S3:**Pareto chart of standardized effects (A) and normal probability plot (B) for number of beads per µm^2^ for FA.

**A**

**B**

**Figure S4:** Main effects plot for average fiber diameter (A) and number of beads per µm^2^ (B) for FA.

**A**

**B**


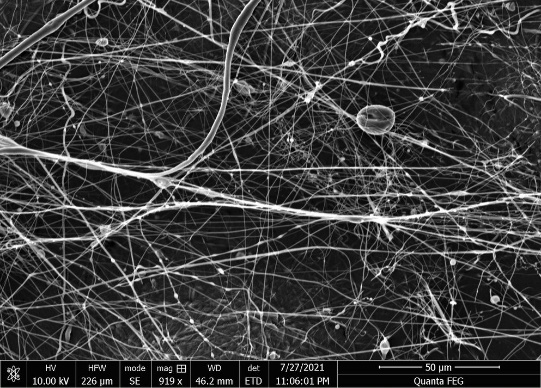

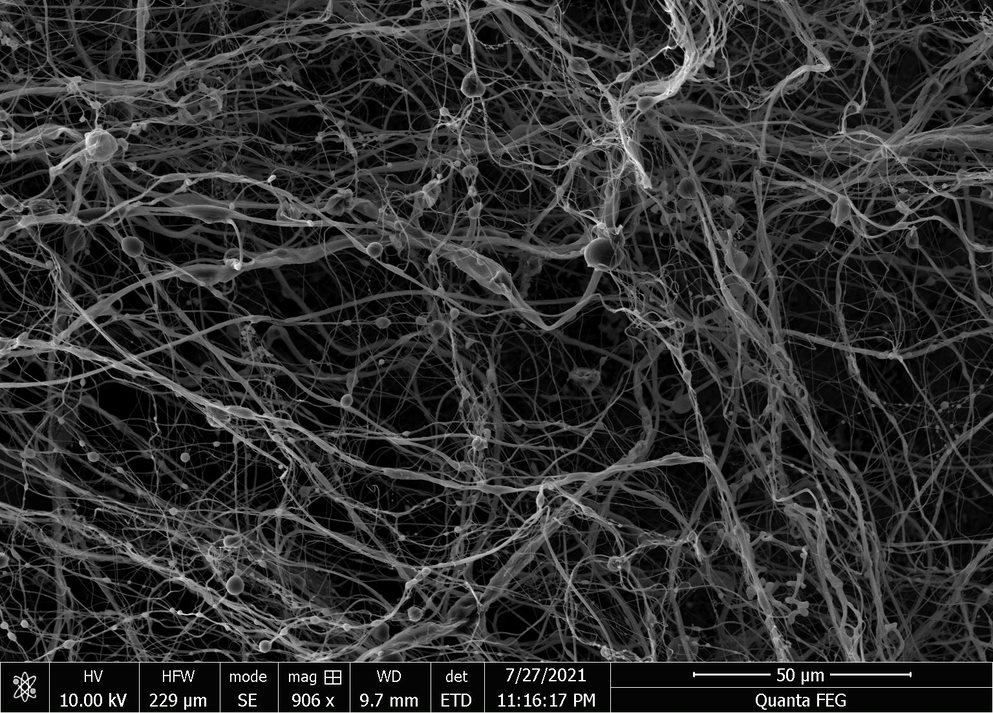

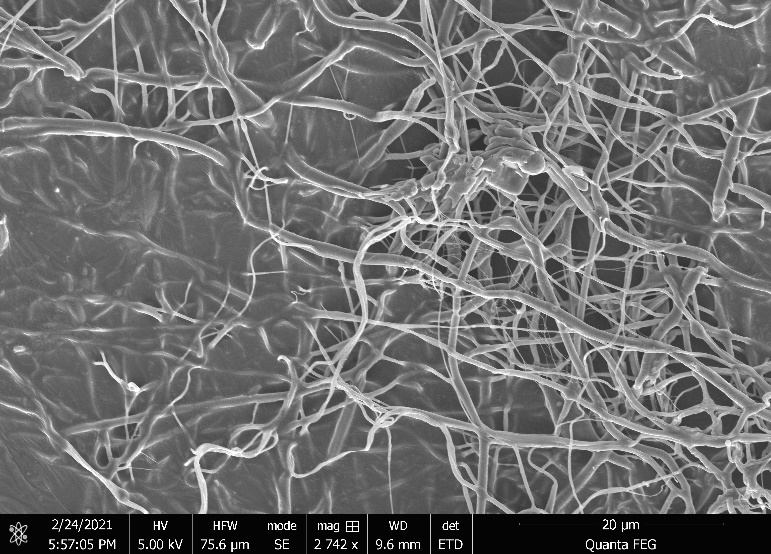

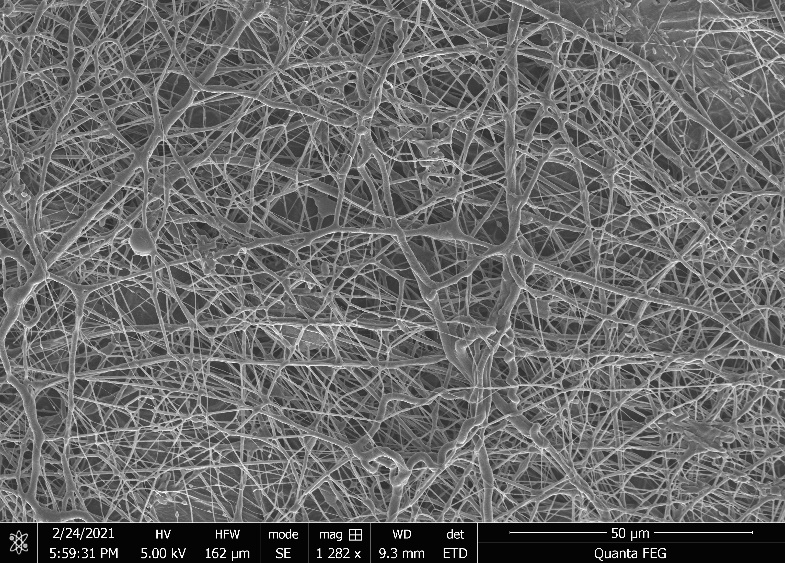


**1**

**2**

**3**

**4**


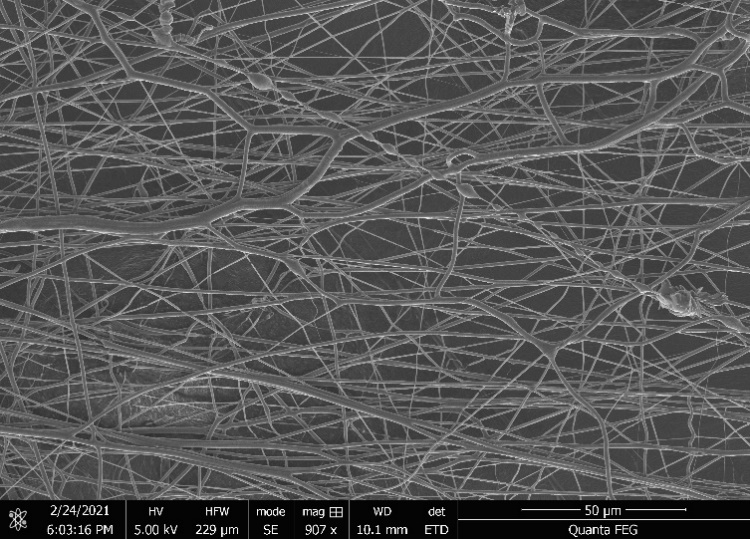

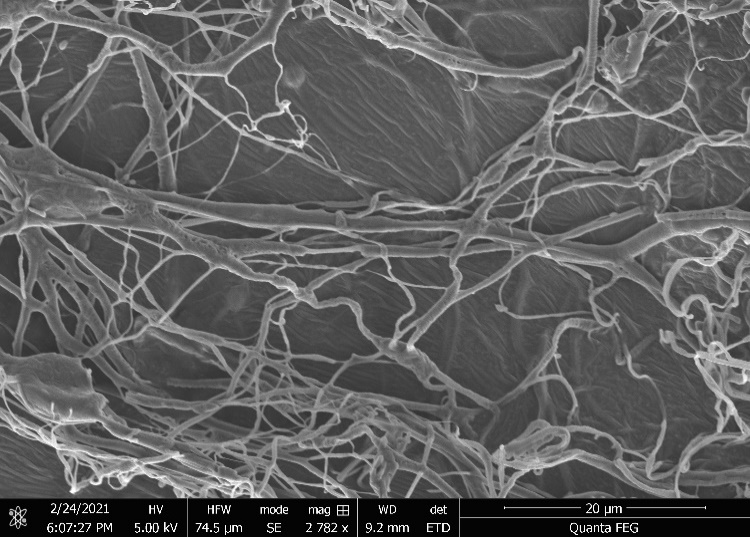

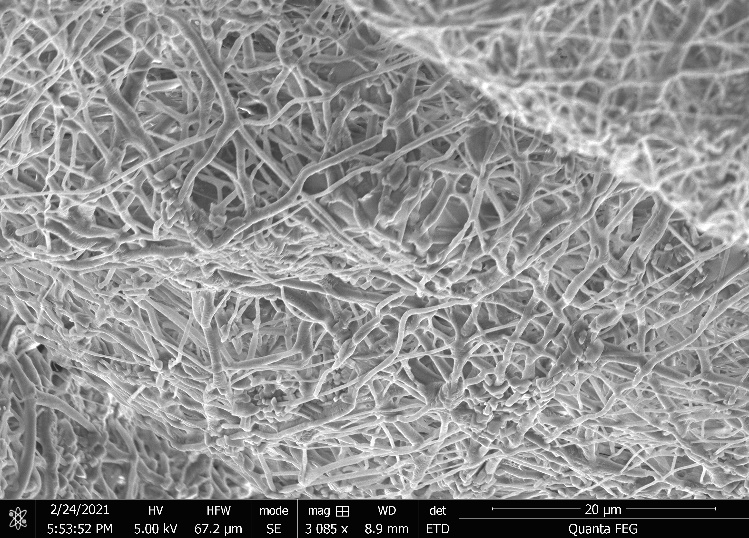


**5**

**6**

**7**

**Figure S5:** SEM images for central composite design runs (1-7) for FA fibers.

**13**

**12**

**10**

**11**


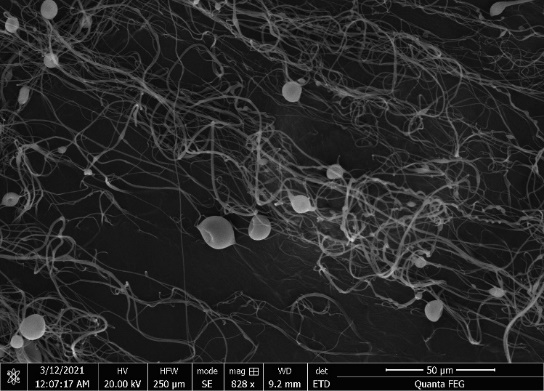


**8**


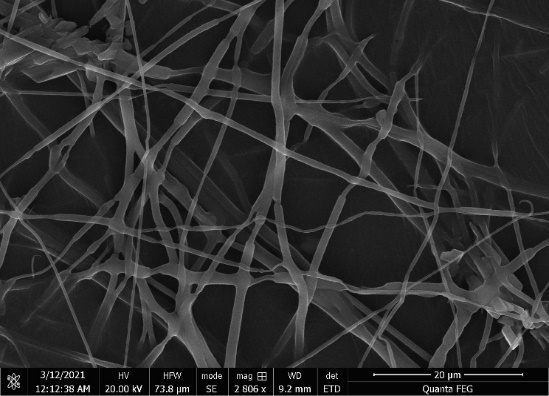


**9**


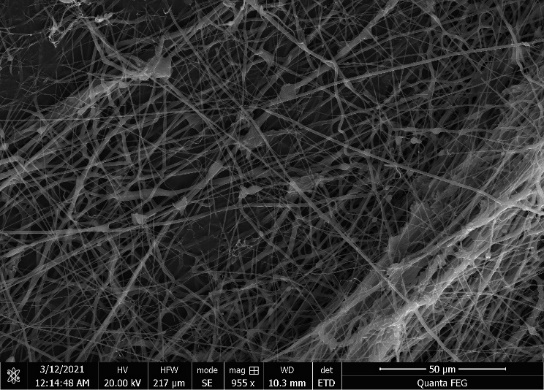

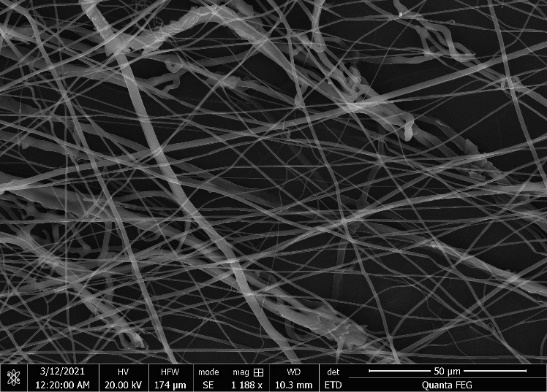

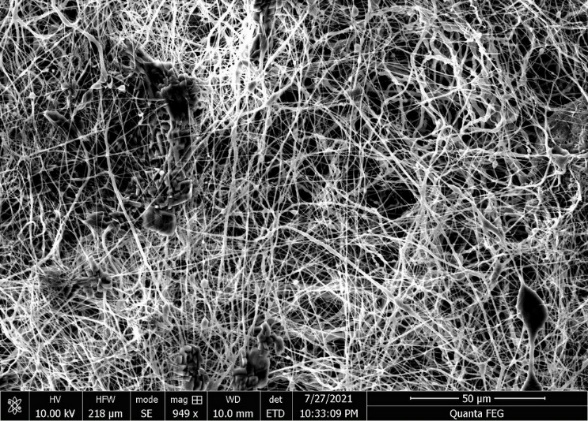

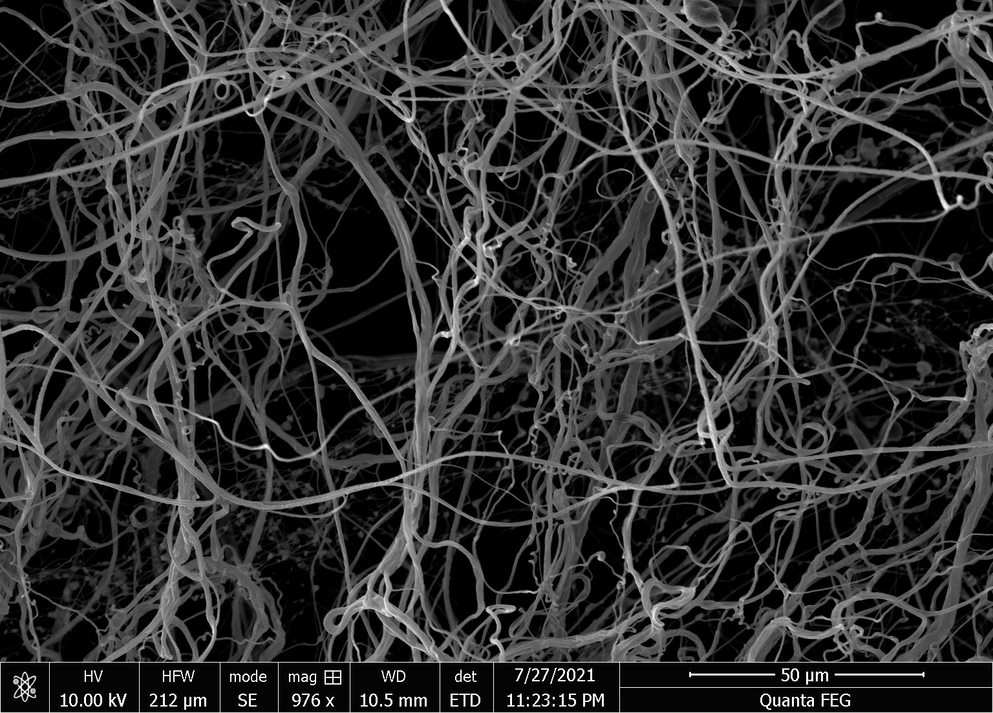


**Figure S6:** SEM images for central composite design runs (8-13) for FA fibers.

**Figure S7:** Residual plots for FA average fiber diameter.

In the normal probability plot, residual points form straight line, thus they are normally distributed. Residual versus fits plot showed random distribution of residuals on both sides of 0, therefore, there is no evidence of non-constant variance. Histogram of residuals plot revealed a normal distribution of residuals, while residual versus order plot showed random distribution of residuals around the center line, revealing that the residuals are independent from one another.

**Figure S8:** Main effects plot for PCL concentration, FA and PAM concentration on average fiber diameter.

**Figure S9:** Contour plot for PCL concentration and FA/PAM concentration vs average fiber diameter.


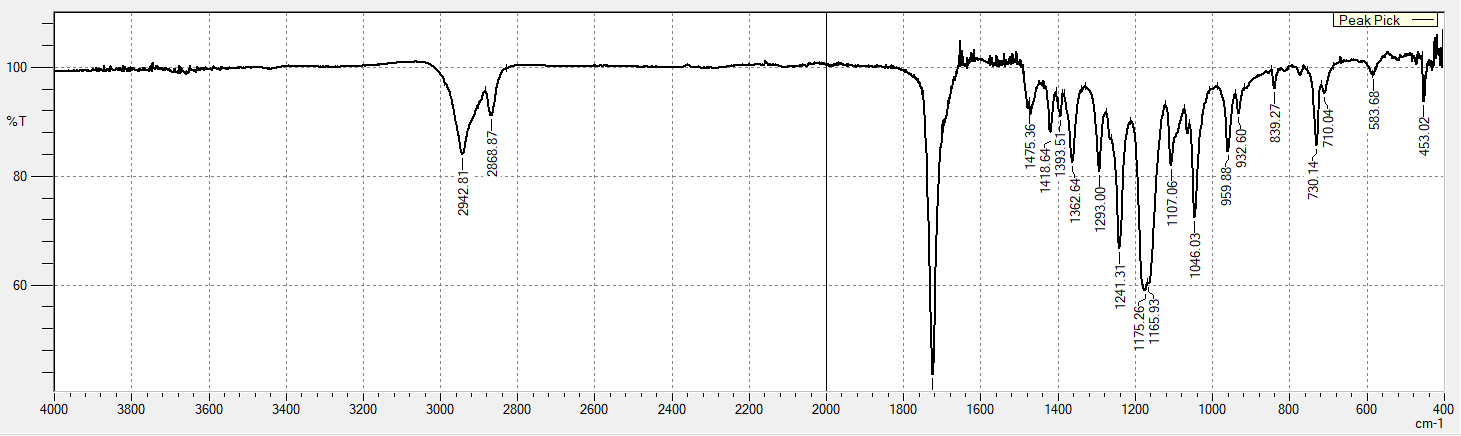


**A**

PCL


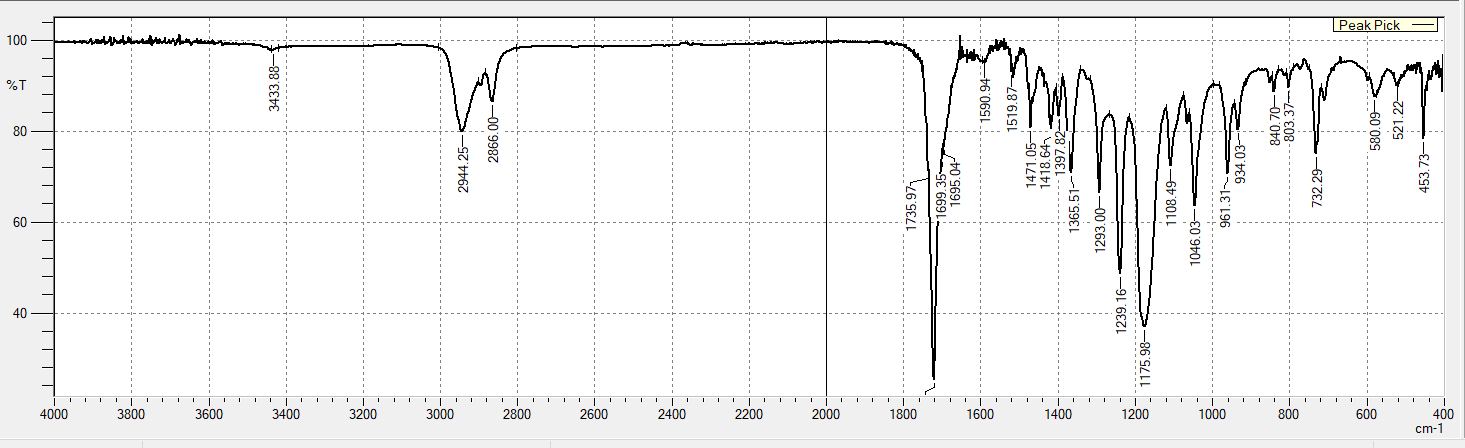


**B**

MI fibers for FA


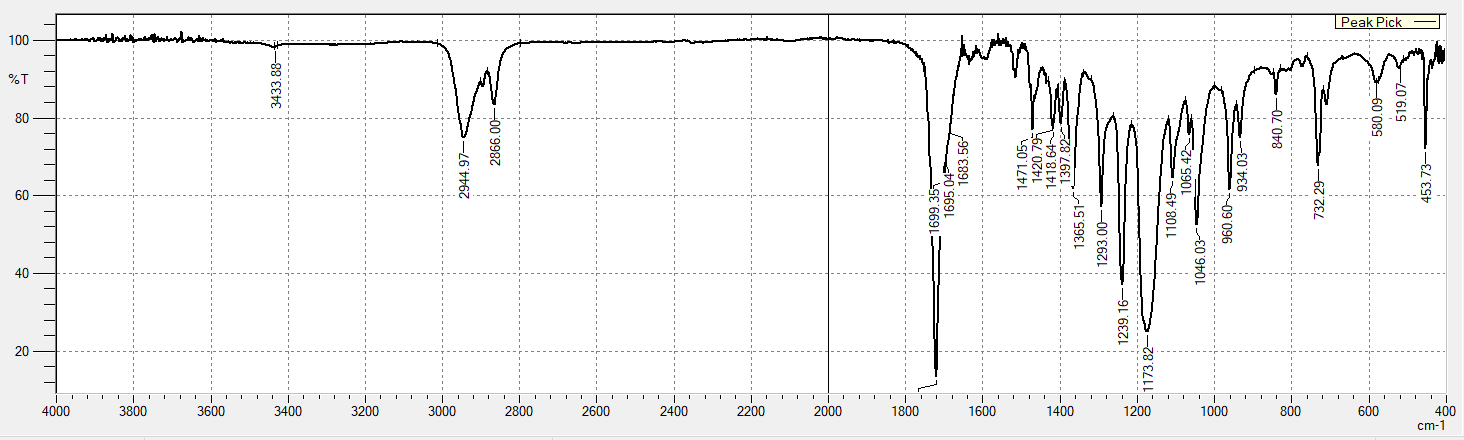


**C**

Control fibers for FA


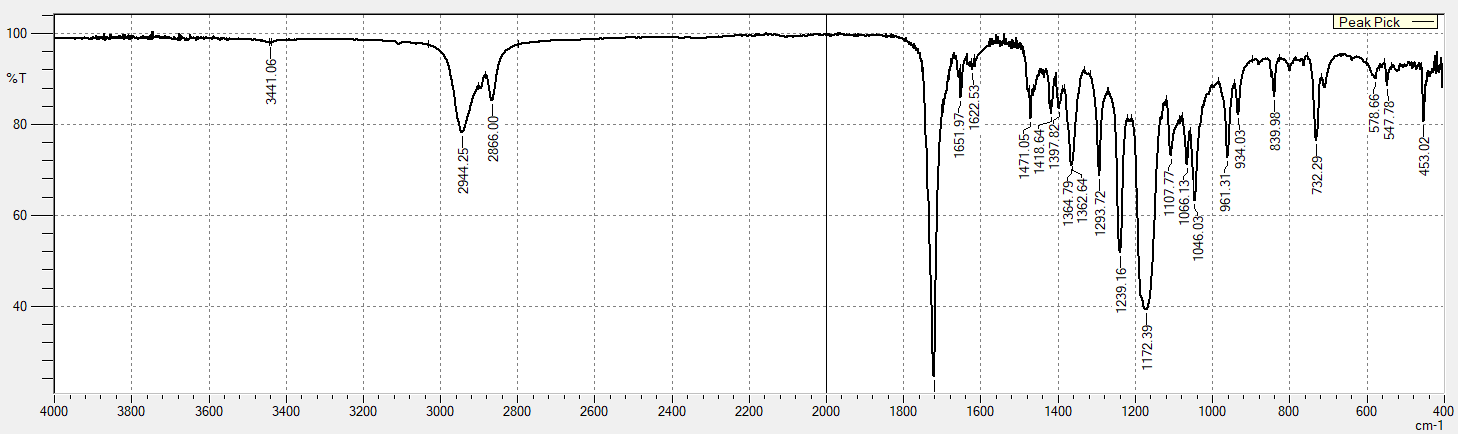


**D**

MI fibers for khellin


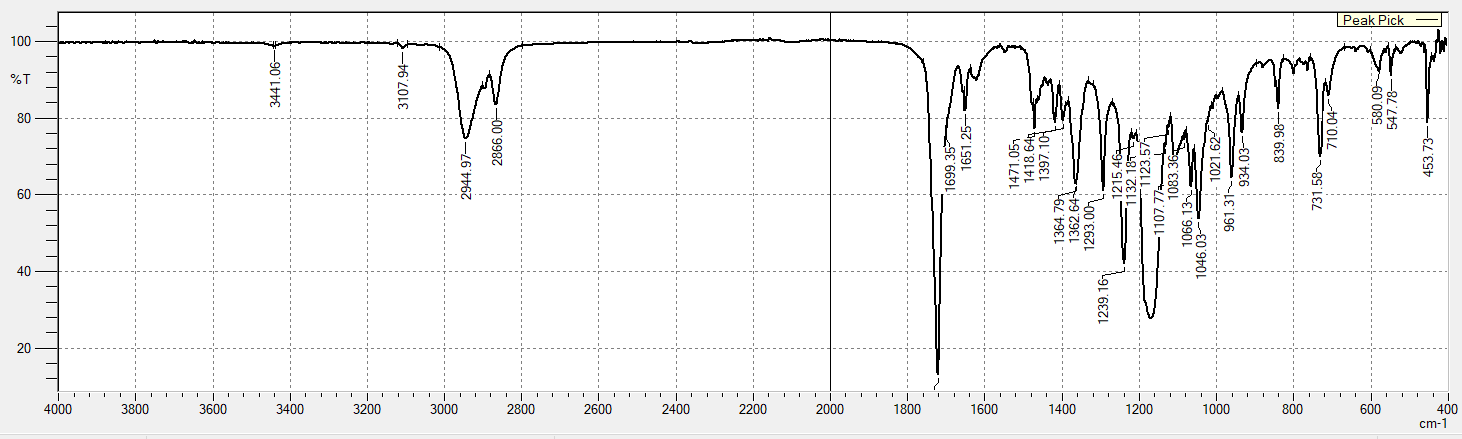


**E**

Control fibers for khellin

**Figure S10**: FTIR spectra for (A) PCL, (B) FA MI fiber, (C) FA control fibers, (D), Khellin MI fibers and (E) Khellin control fibers.

**6**

**4**

**2**


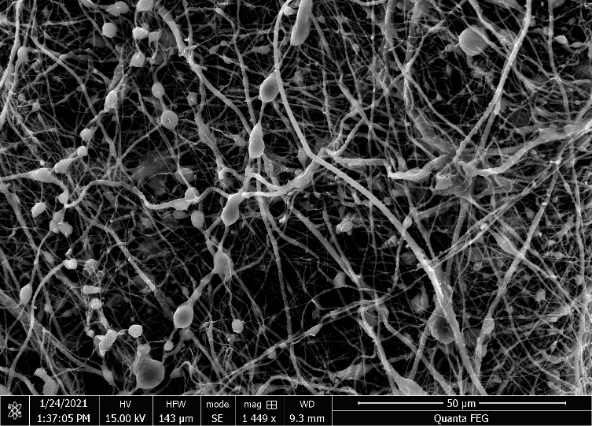

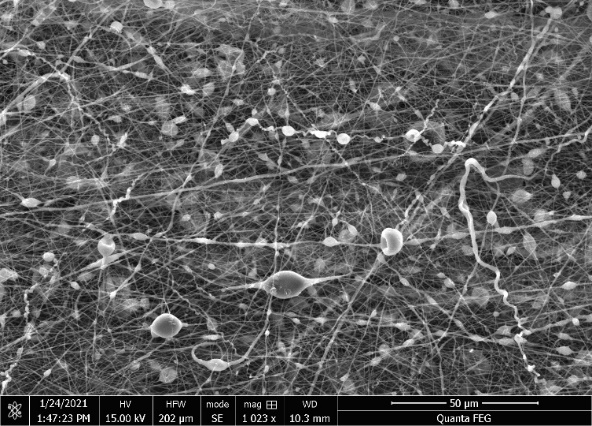

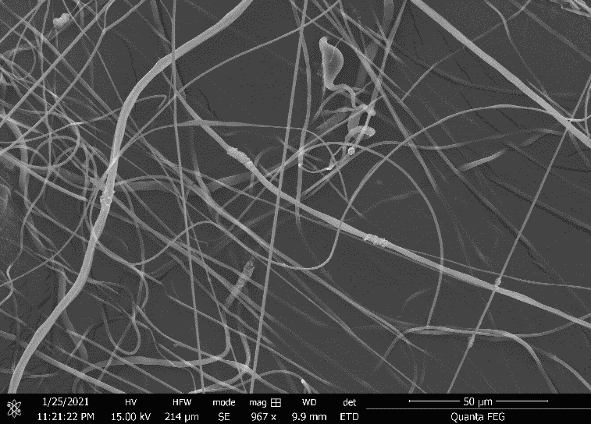


**1**


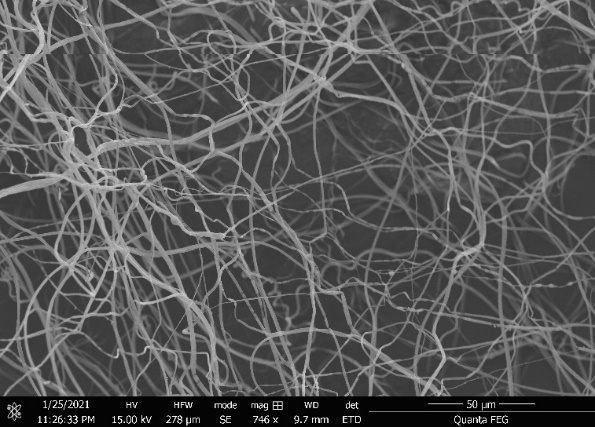

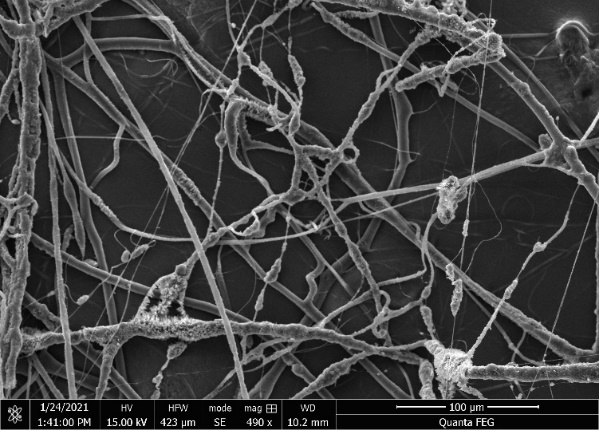

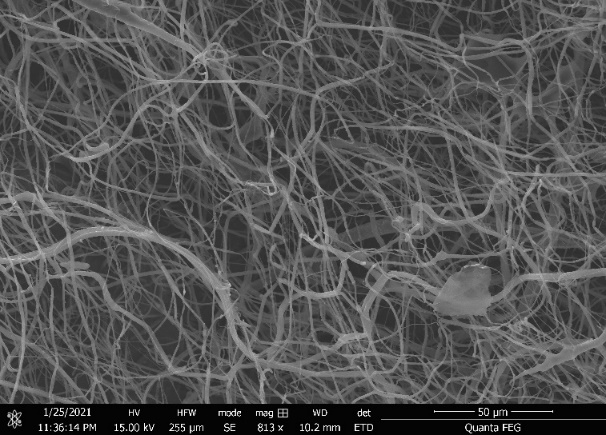

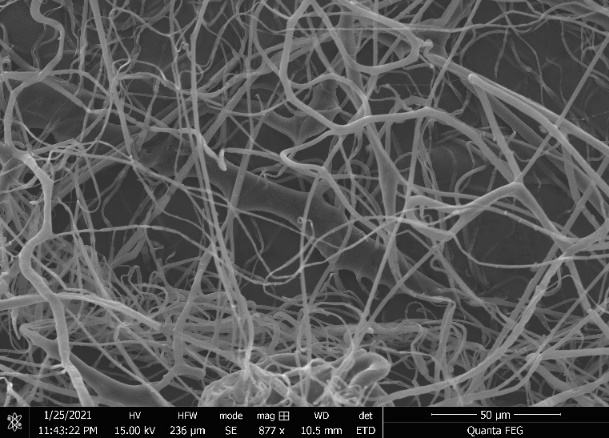

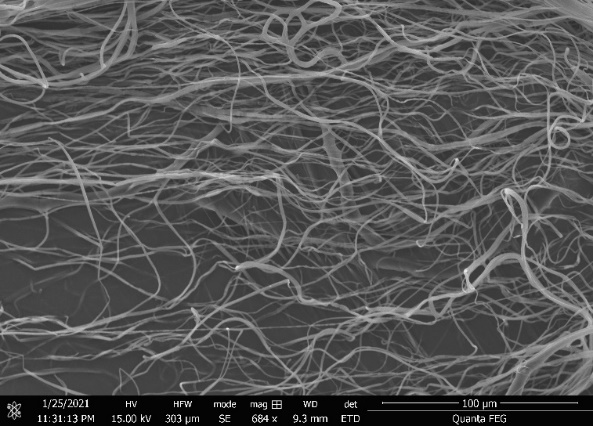


**5**

**8**

**7**

**Figure S11:** SEM Images of Trials 1-8 for Fractional Factorial Design for Khellin.

**3**

**Figure S12:** Pareto chart of standardized effects (A) and normal probability plot (B) for average fiber diameter for khellin.

**Figure S13:** Pareto chart of standardized effects (A) and normal probability plot (B) for average number of beads per µm^2^ for khellin fibers.

**Figure S14:** Main effects plot for average fiber diameter (A) and number of beads per number of beads per µm^2^ for khellin fibers.


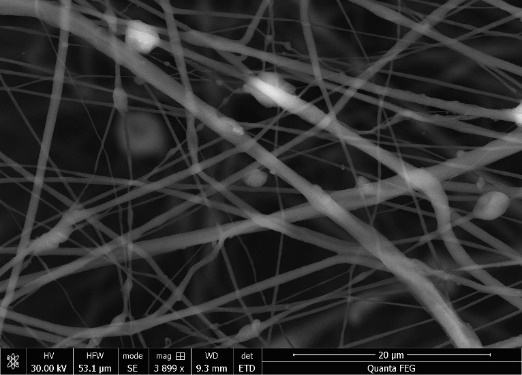

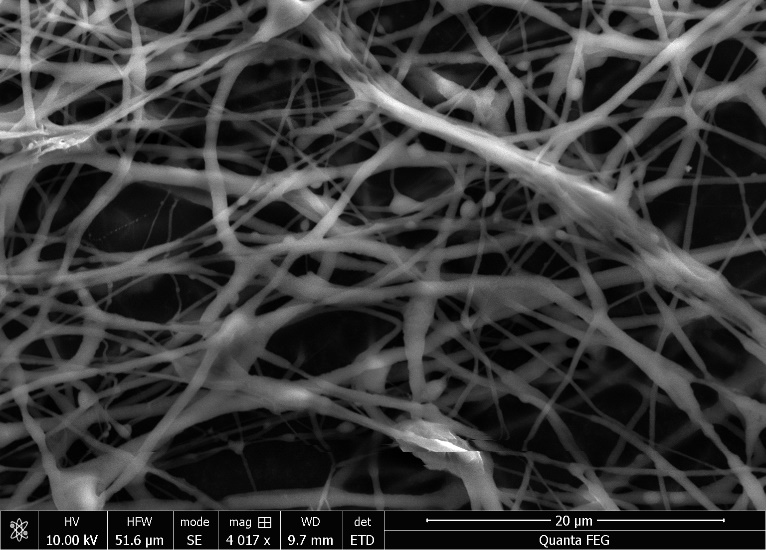

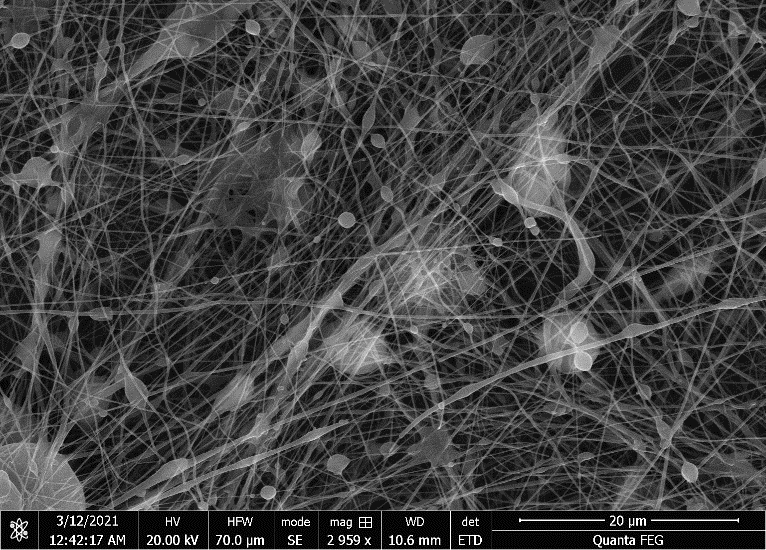

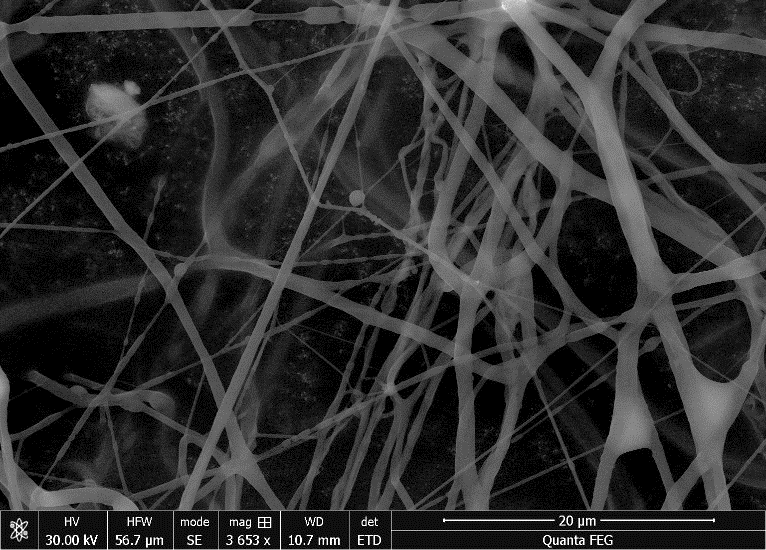


**1**

**2**

**3**

**4**

**7**

**8**


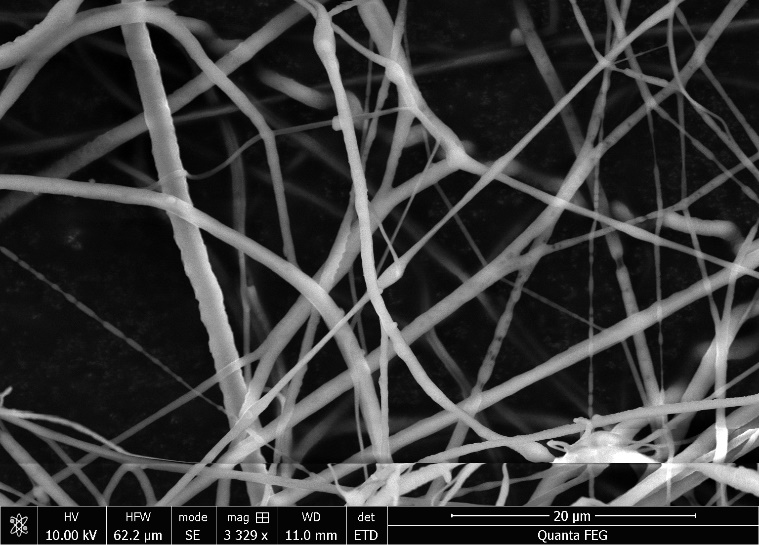

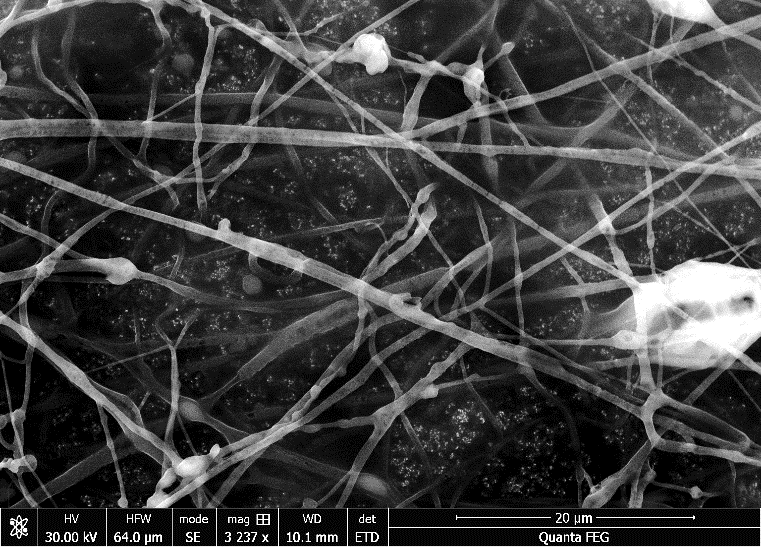

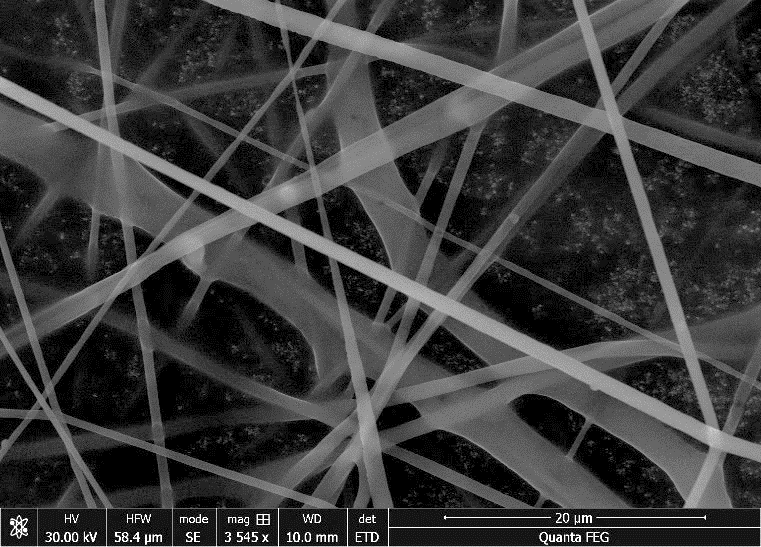

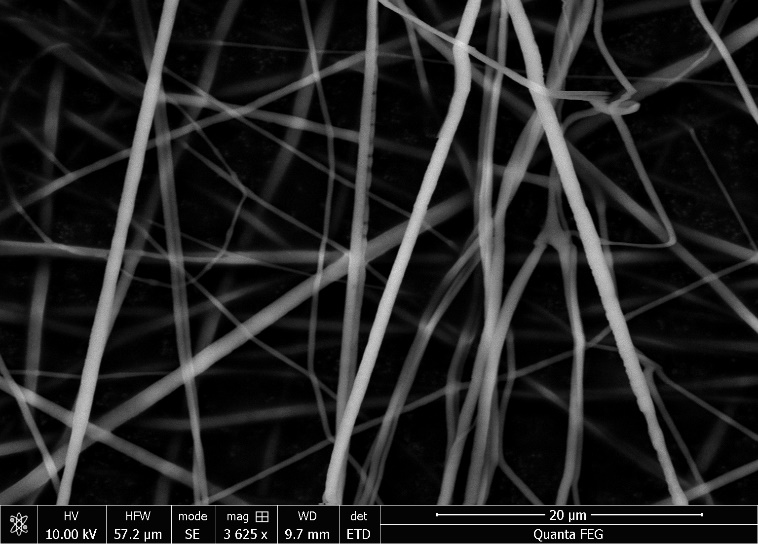


**5**

**6**

**Figure S15**: SEM images for central composite design runs 1-8 for khellin fibers.


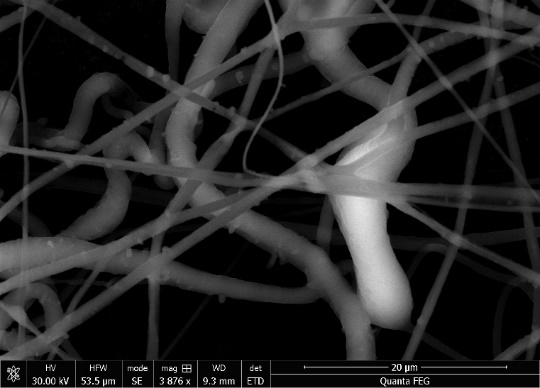

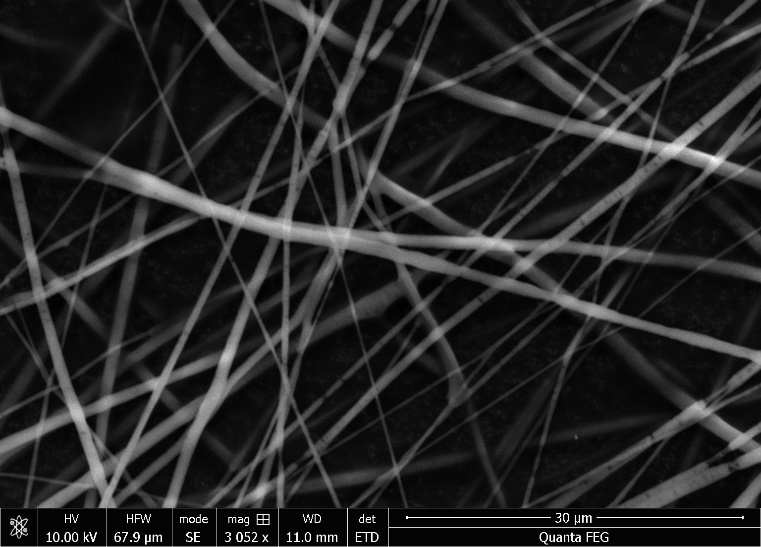

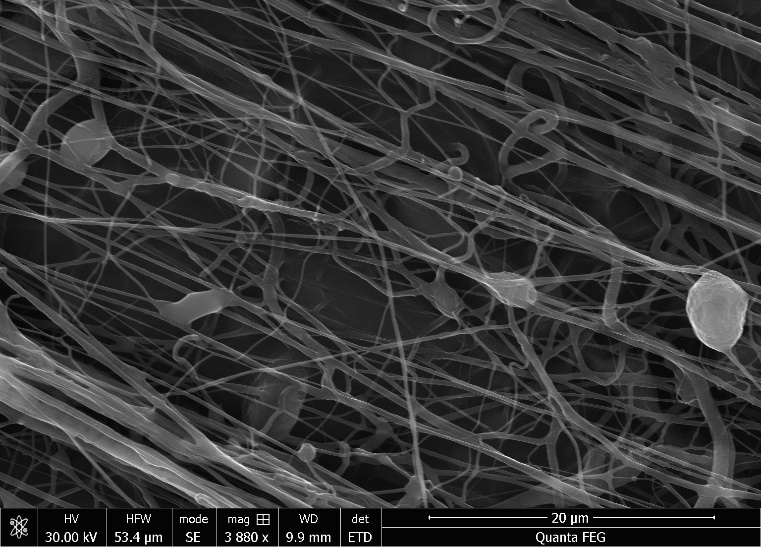

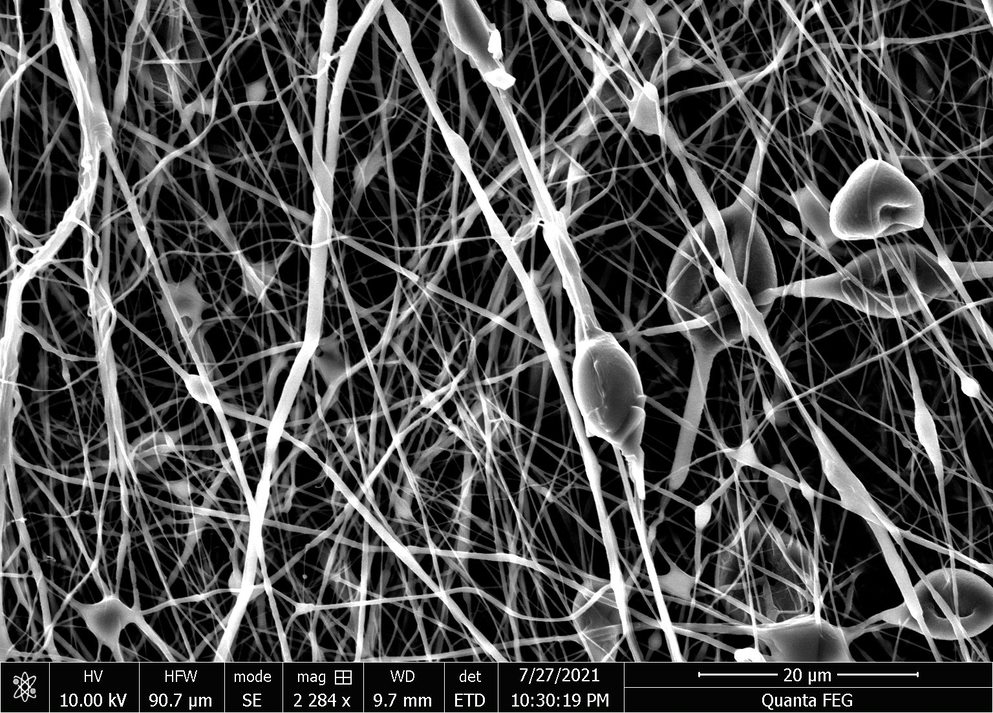


**9**

**10**

**11**

**12**


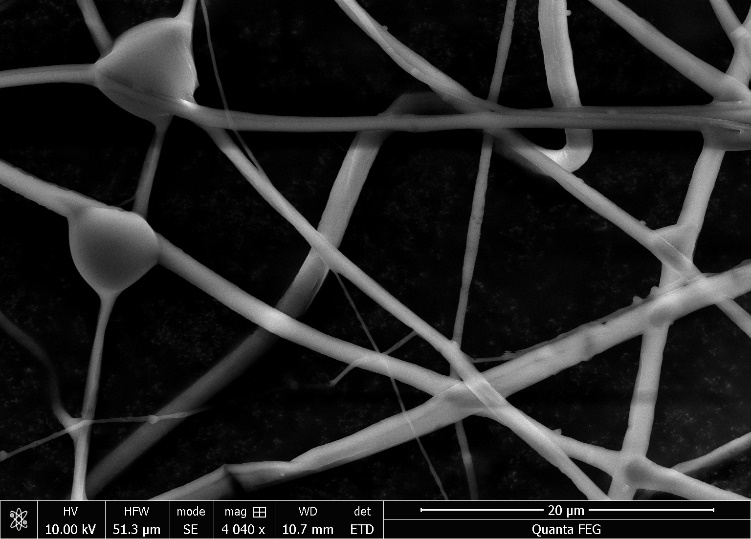



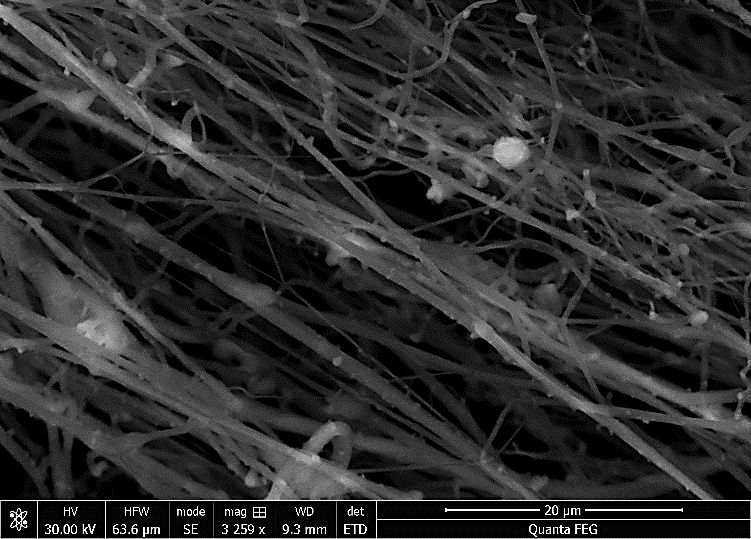

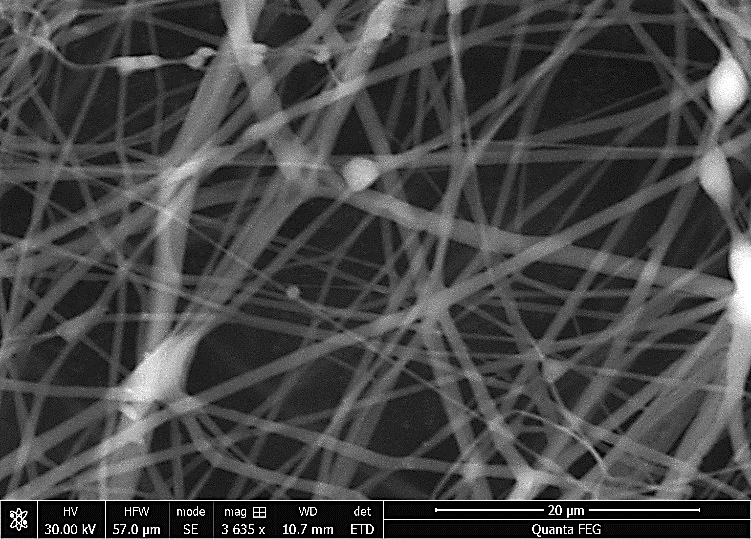


**13**

**14**

**15**

**16**

**Figure S16:** SEM images for central composite design runs 9 - 16 for khellin fibers.


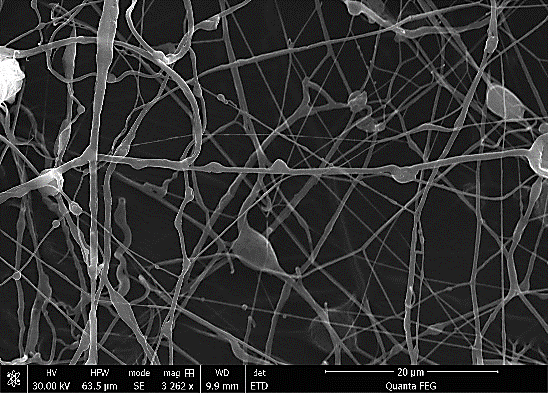

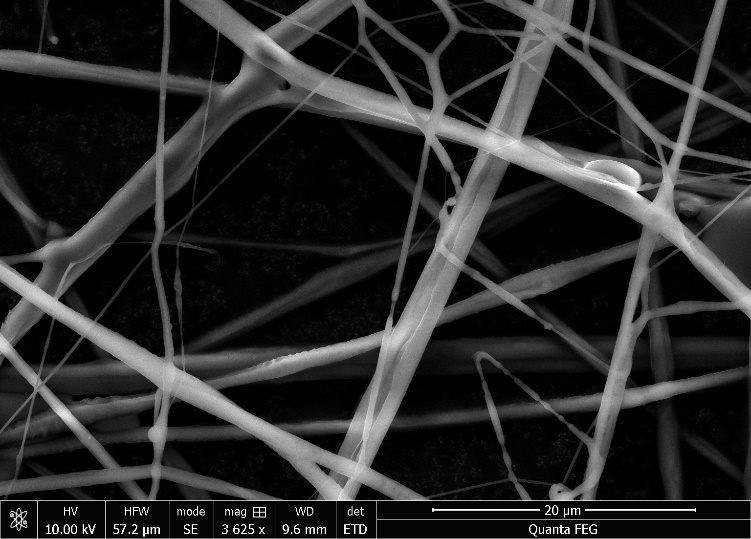

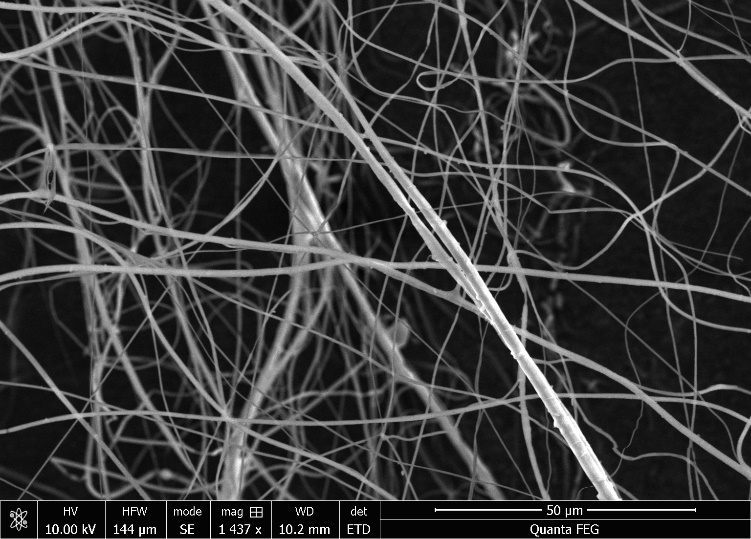

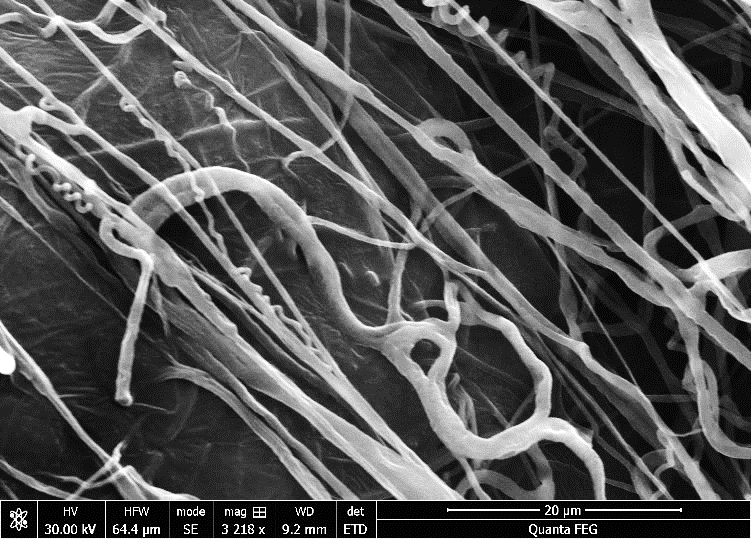


**17**

**18**

**19**

**20**

**Figure S17:** SEM images for central composite design runs 17 - 20 for khellin fibers.

**Figure S18:** Residual plots for khellin average fiber diameter.

In the normal probability plot, residual points form a straight line and are normally distributed. Residual versus fits plot shows a random distribution of residuals on both sides of 0 indicating that there is no evidence of non-constant variance. Histogram of residuals plot shows a normal distribution of residuals. Finally, residual versus order plot showed random distribution of residuals around the central line.

**Figure S19:** Main effects plot for PCL concentration, khellin/PAM concentration and applied voltage on average fiber diameter.

**Figure S20:** Contour plot for khellin average fiber diameter.

**Table S1:** Average fiber diameter and number of beads per µm^2^ for fractional factorial design runs for ferulic acid.

| **Run Order** | **Average Fiber Diameter (nm) ± SD** | **Number of beads per µm^2^**  **± SD** |
| --- | --- | --- |
| **1** | 260 ± 95 | 0.00678 ± 0.0002 |
| **2** | 380 ± 128 | 0.0101 ± 0.0003 |
| **3** | 1252 ± 252 | 0 |
| **4** | 648 ± 294 | 0.0150 ± 0.00007 |
| **5** | 1228 ± 315 | 0 |
| **6** | 1001 ± 251 | 0 |
| **7** | 1263 ± 467 | 0 |
| **8** | 653 ± 378 | 0.008877 ± 0.0001 |

**Table S2:** Regression model summary for ferulic acid fibers.

| **Model Term** | | **p-value** | |
| --- | --- | --- | --- |
| **Constant** | | 0.026 | |
| **PCL concentration** | | 0.005 | |
| **FA and PAM concentration** | | 0.019 | |
| **PCL concentration^2^** | | 0.006 | |
| **PCL concentration× FA and PAM concentration** | | 0.008 | |
| S  44.9466 | R^2^  98.04 % | R^2^ (adjusted)  97.07 % | R^2^ (predicted)  94.74 % |

**Table S3:** Average fiber diameter and number of beads per µm2 for fractional factorial design runs for khellin

| **Run Order** | **Average Fiber Diameter (nm)**  **± SD** | **Number of beads per µm^2^**  **± SD** |
| --- | --- | --- |
| **1** | 553 ± 285 | 0.00484 ± 0.0007 |
| **2** | 404 ± 194 | 0.00346 ± 0.0009 |
| **3** | 1239 ± 128 | 0 |
| **4** | 698 ± 201 | 0 |
| **5** | 2250 ± 596 | 0.000102 ± 0.0003 |
| **6** | 1186 ± 265 | 0.0000647 ± 0.000001 |
| **7** | 1426 ± 312 | 0 |
| **8** | 1245 ± 298 | 0 |

**Table S4:** Regression model summary for khellin fibers.

| **Model Term** | | **p-value** | |
| --- | --- | --- | --- |
| **Constant** | | 0.010 | |
| **PCL concentration** | | 0.000 | |
| **Khellin and PAM concentration** | | 0.026 | |
| **Applied voltage** | | 0.045 | |
| **Applied voltage^2^** | | 0.047 | |
| S  78.7654 | R^2^  96.69 % | R^2^ (adjusted)  95.80 % | R^2^ (predicted)  93.23 % |

**Table S5:** MTT results for non-medicated fibers vs MI FA fibers.

| Non-medicated control FA fibers | | | | | | | | |
| --- | --- | --- | --- | --- | --- | --- | --- | --- |
| Concentration (μg/ml) | 500 | 250 | 125 | 62.5 | 50 | 25 | 12.5 | 6.25 |
| % Cell Viability | 154.154 | 160.475 | 110.369 | 96.664 | 100.185 | 115.432 | 109.092 | 88.662 |
| St. Dev. | 1.5610 | 6.671 | 8.860 | 23.135 | 0 | 0 | 11.436 | 9.480 |
| MI FA fibers | | | | | | | | |
| Concentration (μg/ml) | 500 | 250 | 125 | 62.5 | 50 | 25 | 12.5 | 6.25 |
| % Cell Viability | 50.414 | 91.739 | 153.478 | 94.385 | 109.796 | 90.225 | 96.694 | 91.119 |
| St. Dev. | 3.994 | 15.390 | 21.867 | 10.620 | 0 | 0 | 4.595 | 2.330 |

**Table S6:** MTT results for non-medicated fibers vs MI khellin fibers.

| Non-medicated control khellin fibers | | | | | | | | |
| --- | --- | --- | --- | --- | --- | --- | --- | --- |
| Concentration (μg/ml) | 500 | 250 | 125 | 62.5 | 50 | 25 | 12.5 | 6.25 |
| % Cell Viability | 154.155 | 160.475 | 110.360 | 96.665 | 100.185 | 115.433 | 109.093 | 88.662 |
| St. Dev. | 1.561 | 6.671 | 8.860 | 23.135 | 0 | 0 | 11.436 | 9.480 |
| MI khellin fibers | | | | | | | | |
| Concentration (μg/ml) | 500 | 250 | 125 | 62.5 | 50 | 25 | 12.5 | 6.25 |
| % Cell Viability | 60.440 | 77.769 | 149.418 | 111.582 | 105.127 | 110.650 | 101.589 | 104.237 |
| St. Dev. | 5.031 | 8.382 | 17.506 | 12.146 | 0 | 0 | 4.303 | 2.200 |

**Table S7:** Drug permeation results for ferulic acid fibers over 24 hours.

| **Time (h)** | **% FA permeated** |
| --- | --- |
| **0.25** | 0.00% |
| **0.5** | 0.00% |
| **1** | 0.00% |
| **2** | 0.05 ± 0.0004% |
| **4** | 0.36 ± 0.005% |
| **6** | 2.38 ± 0.014% |
| **8** | 5.03 ± 0.030% |
| **24** | 12.71 ± 0.053% |

**Table S8:** Drug permeation results for khellin fibers over 24 hours.

| **Time (h)** | **% Khellin permeated** |
| --- | --- |
| **0.25** | 0.00% |
| **0.5** | 4.19 ± 0.021% |
| **1** | 5 .11± 0.025% |
| **2** | 5.70 ± 0.042% |
| **4** | 6.14 ± 0.041% |
| **6** | 7.76 ± 0.056% |
| **8** | 9.19 ± 0.059% |
| **24** | 22.99 ± 0.041% |

**UHPLC Method for Ferulic Acid Quantification**

The UHPLC-UV method was validated in terms of linearity, LOD, LOQ and precision (inter- and intra-day). A linear calibration curve was obtained over the concentration range 0.01-0.4 mM. The linear correlation equation obtained was y = 180632x - 616.44 with R² = 0.995.

The precision was evaluated in terms of %RSD of peak areas and RTs. The intra-day precision was tested by injecting three different concentrations (0.015, 0.15 and 0.3 mM) separately in triplicate on the same day, while the inter-day precision was tested by injecting the same concentration in triplicate on three consecutive days. The %RSD for peak areas were within the range 0.97 – 1.84 for intra-day assays and within 0.43 – 1.36 for inter-day assays. As for RTs, the %RSD ranges were 0.27 – 0.74 and 0.64 – 1.33 for intra- and inter-day assays respectively **(Table S9)**. The method showed LOD of 0.00163 mM and LOQ of 0.00493.

**UHPLC Method for Khellin Quantification**

For Khellin, a linear calibration curve was obtained over the concentration range of 0.01 – 0.4 mM. The linear correlation equation was y = 253368x – 1953 with R² = 0.9962

The precision was evaluated in terms of %RSD of peak areas and RTs. The intra-day precision was tested by injecting three different concentrations (0.015, 0.15 and 0.3 mM) separately in triplicate on the same day, while the inter-day precision was tested by injecting the same concentration in triplicate on three consecutive days. The %RSD for peak areas were within the range 1.57 – 2.43 for intra-day assays and within 1.51 – 4.12 for inter-day assays. As for RTs, the %RSD ranges were 0.16 – 0.89 and 1.51 – 4.12 for intra- and inter-day assays respectively **(Table S10)**. The method has revealed low LOD of 0.00217 mM and low LOQ of 0.00658 mM.

Four residual plots were obtained; normal probability of residuals, residuals versus fits, histogram of residuals and residuals versus order plots **(Figure S7)**. Details were added in supplementary data. In the normal probability plot, residual points form straight line, thus they are normally distributed. Residual versus fits plot showed random distribution of residuals on both sides of 0, therefore, there is no evidence of non-constant variance. Histogram of residuals plot revealed a normal distribution of residuals, while residual versus order plot showed random distribution of residuals around the center line, revealing that the residuals are independent from one another.

**Table S9:** Intra-day and inter-day precision assay for UHPLC-UV measurements for FA.

| **Concentration**  **(mM)** | **Intra-day RSD%** | | **Inter-day RSD%** | |
| --- | --- | --- | --- | --- |
|  | **Peak area** | **Retention time** | **Peak area** | **Retention time** |
| **0.015** | 1.84 | 0.27 | 1.36 | 0.64 |
| **0.15** | 0.97 | 0.71 | 1.27 | 1.33 |
| **0.3** | 1.22 | 0.74 | 0.43 | 0.99 |

**Table S10:** Intra-day and inter-day precision assay for UHPLC-UV measurements for khellin.

| **Concentration**  **(mM)** | **Intra-day RSD%** | | **Inter-day RSD%** | |
| --- | --- | --- | --- | --- |
|  | **Peak area** | **Retention time** | **Peak area** | **Retention time** |
| **0.015** | 1.72 | 0.31 | 2.26 | 1.51 |
| **0.15** | 2.43 | 0.16 | 3.97 | 2.30 |
| **0.3** | 1.57 | 0.89 | 3.91 | 4.12 |
